# Gut microbial carbohydrate metabolism contributes to insulin resistance

**DOI:** 10.1038/s41586-023-06466-x

**Published:** 2023-08-30

**Authors:** Tadashi Takeuchi, Tetsuya Kubota, Yumiko Nakanishi, Hiroshi Tsugawa, Wataru Suda, Andrew Tae-Jun Kwon, Junshi Yazaki, Kazutaka Ikeda, Shino Nemoto, Yoshiki Mochizuki, Toshimori Kitami, Katsuyuki Yugi, Yoshiko Mizuno, Nobutake Yamamichi, Tsutomu Yamazaki, Iseki Takamoto, Naoto Kubota, Takashi Kadowaki, Erik Arner, Piero Carninci, Osamu Ohara, Makoto Arita, Masahira Hattori, Shigeo Koyasu, Hiroshi Ohno

**Affiliations:** 1 Laboratory for Intestinal Ecosystem, RIKEN Center for Integrative Medical Sciences (IMS), Yokohama, Japan; 2 Intestinal Microbiota Project, Kanagawa Institute of Industrial Science and Technology, Kawasaki, Japan; 3 Department of Diabetes and Metabolic Diseases, Graduate School of Medicine, The University of Tokyo, Tokyo, Japan; 4 Division of Diabetes and Metabolism, The Institute for Medical Science Asahi Life Foundation, Tokyo, Japan; 5 Department of Clinical Nutrition, National Institutes of Biomedical Innovation, Health and Nutrition (NIBIOHN), Tokyo, Japan; 6 Metabolome Informatics Research Team, RIKEN Center for Sustainable Resource Science (CSRS), Yokohama, Japan; 7 Laboratory for Metabolomics, RIKEN Center for Integrative Medical Sciences (IMS), Yokohama, Japan; 8 Graduate School of Medical Life Science, Yokohama City University, Yokohama, Japan; 9 Department of Biotechnology and Life Science, Tokyo University of Agriculture and Technology, Tokyo, Japan; 10 Laboratory for Microbiome Sciences, RIKEN Center for Integrative Medical Sciences (IMS), Yokohama, Japan; 11 Laboratory for Applied Regulatory Genomics Network Analysis, RIKEN Center for Integrative Medical Sciences (IMS), Yokohama, Japan; 12 Laboratory for Integrative Genomics, RIKEN Center for Integrative Medical Sciences (IMS), Yokohama, Japan; 13 Department of Applied Genomics, Kazusa DNA Research Institute, Kisarazu, Japan; 14 Laboratory for Developmental Genetics, RIKEN Center for Integrative Medical Sciences (IMS), Yokohama, Japan; 15 Laboratory for Integrated Cellular Systems, RIKEN Center for Integrative Medical Sciences (IMS), Yokohama, Japan; 16 Institute for Advanced Biosciences, Keio University, Fujisawa, Japan; 17 Department of Biological Sciences, Graduate School of Science, The University of Tokyo, Tokyo, Japan; 18 Department of Cardiovascular Medicine, The University of Tokyo, Tokyo, Japan; 19 Development Bank of Japan, Tokyo, Japan; 20 Center for Epidemiology and Preventive Medicine, The University of Tokyo Hospital, Tokyo, Japan; 21 International University of Health and Welfare, Tokyo, Japan; 22 Department of Metabolism and Endocrinology, Tokyo Medical University Ibaraki Medical Center, Ami Town, Japan; 23 Toranomon Hospital, Tokyo, Japan; 24 Laboratory for Transcriptome Technology, RIKEN Center for Integrative Medical Sciences (IMS), Yokohama, Japan; 25 Fondazione Human Technopole, Milan, Italy; 26 Division of Physiological Chemistry and Metabolism, Graduate School of Pharmaceutical Sciences, Keio University, Tokyo, Japan; 27 Human Biology-Microbiome-Quantum Research Center (WPI-Bio2Q), Keio University, Tokyo, Japan; 28 Laboratory for Immune Cell Systems, RIKEN Center for Integrative Medical Sciences (IMS), Yokohama, Japan

**Keywords:** Metabolomics, Next-generation sequencing, Pre-diabetes, Microbiome, Metagenomics

## Abstract

Insulin resistance is the primary pathophysiology underlying metabolic syndrome and type 2 diabetes^1,2^. Previous metagenomic studies have described the characteristics of gut microbiota and their roles in metabolizing major nutrients in insulin resistance^3–9^. In particular, carbohydrate metabolism of commensals has been proposed to contribute up to 10% of the host’s overall energy extraction^10^, thereby playing a role in the pathogenesis of obesity and prediabetes^3,4,6^. Nevertheless, the underlying mechanism remains unclear. Here we investigate this relationship using a comprehensive multi-omics strategy in humans. We combine unbiased faecal metabolomics with metagenomics, host metabolomics and transcriptomics data to profile the involvement of the microbiome in insulin resistance. These data reveal that faecal carbohydrates, particularly host-accessible monosaccharides, are increased in individuals with insulin resistance and are associated with microbial carbohydrate metabolisms and host inflammatory cytokines. We identify gut bacteria associated with insulin resistance and insulin sensitivity that show a distinct pattern of carbohydrate metabolism, and demonstrate that insulin-sensitivity-associated bacteria ameliorate host phenotypes of insulin resistance in a mouse model. Our study, which provides a comprehensive view of the host–microorganism relationships in insulin resistance, reveals the impact of carbohydrate metabolism by microbiota, suggesting a potential therapeutic target for ameliorating insulin resistance.

## Abstract (web summary)

Faecal carbohydrates, particularly host-accessible monosaccharides, are increased in individuals with insulin resistance and are associated with microbial carbohydrate metabolisms and host inflammatory cytokines.

## Main

**Extended Data Fig. 1:**
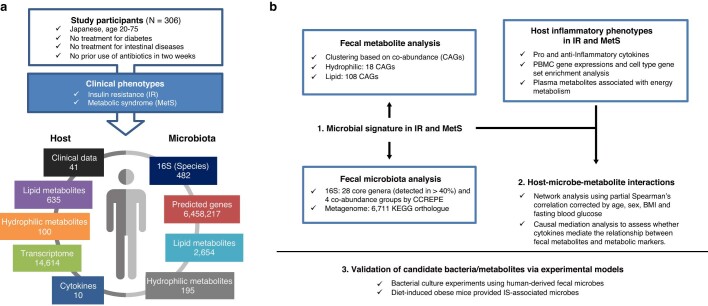
Overview of multi-omics analysis and data. **a**, Individuals without a prior diagnosis of diabetes, diabetic medications, or intestinal diseases were included (n = 306). Insulin resistance (IR) and metabolic syndrome (MetS) were the main clinical phenotypes. To evaluate the host-microbe relationship, we collected 1) host factors: clinical, plasma metabolome, peripheral blood mononuclear cells (PBMC) transcriptome, and cytokine data, and 2) microbial factors: 16S rRNA pyrosequencing, shotgun metagenome, and faecal metabolome. The numbers of elements after quality filtering are shown for each data set. **b**, The multi-omics analysis workflow. To identify the microbes that affect metabolic phenotypes, we first analysed the phenotype-associated metabolomic signatures by binning metabolites into co-abundance groups (CAGs). Microbial signatures were determined using the 16S and metagenomic datasets, and their associations with metabolites were analysed. To gain insight into the host-microbe relationship, the associations among faecal metabolites/microbes and host plasma metabolites, cytokines, and PBMC genes were analysed. We also assessed the mediation effects of plasma cytokines on the relationships between faecal metabolites and metabolic markers. Finally, to validate the effects of candidate metabolites/microbes on metabolic phenotypes, we performed bacterial culture and animal experiments. The associations between clinical phenotypes and omics markers were adjusted by age and sex wherever appropriate.

We analysed 306 individuals (71% male) aged from 20 to 75 years (median age, 61 years), who were recruited during their annual health check-ups (Extended Data Fig. 1a). Individuals diagnosed with diabetes were excluded to avoid any long-lasting effects of hyperglycaemia^5,6^. Consequently, our study included relatively healthy individuals compared with most of the previous metagenomic studies of diabetes and obesity^5–8,11,12^; the median (interquartile range (IQR)) body mass index (BMI) and glycated haemoglobin (HbA1c) were 24.9 kg m^−2^ (22.2–27.1 kg m^−2^) and 5.8% (5.5–6.1%), respectively (Supplementary Table 1). The main clinical phenotype analysed in this study was insulin resistance (IR), which we defined as a homeostatic model assessment of IR (HOMA-IR) score of at least 2.5 (ref. ^13^). We also analysed the associations between faecal metabolites and metabolic syndrome (MetS), an IR-related pathology. The clinical characteristics of IR and MetS largely overlapped except for blood pressure and sex ratio, for which there was no difference between individuals with IR versus normal insulin sensitivity (IS) (Supplementary Table 1). Untargeted metabolomics analysis using two mass spectrometry (MS)-based analytical platforms identified 195 and 100 annotated faecal and plasma hydrophilic metabolites, and 2,654 and 635 annotated faecal and plasma lipid metabolites, respectively (Extended Data Fig. 1a). To identify the overall difference in microbial functions, faecal metabolites and predicted genes were summarized into co-abundance groups (CAGs) and KEGG categories, respectively (Extended Data Fig. 1b). Transcriptomic information of peripheral blood mononuclear cells (PBMCs) was obtained using the cap analysis of gene expression (CAGE) method^14^, which can measure gene expression at the transcription-start-site resolution.

**Fig. 1:**
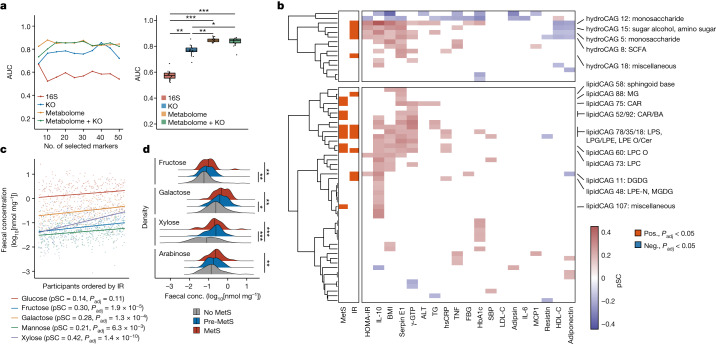
Faecal carbohydrate metabolites are distinctly altered in IR. **a**, Left, the AUC of random forest classifiers was used to predict IR based on genus-level 16S (*n* = 282), metagenome at the KEGG orthologue (KO) level (*n* = 266), faecal metabolome and metagenome (KEGG orthologue) + faecal metabolome (*n* = 266) data. The number of featured markers selected from the datasets increases along the *x* axis. Right, the box plots show the AUC obtained by selected features. Each dot represents an AUC value of a random-forest classifier using a given number of selected features as predictor variables. **b**, CAGs of faecal hydrophilic metabolites (hydroCAG, top) and lipid metabolites (lipidCAG, bottom), and clinical phenotypes and markers (*n* = 282). The two-column heat map on the left represents the associations with the main clinical phenotypes (IR and MetS) analysed using rank-based linear regression, whereas the main heat map shows the partial Spearman’s correlations (pSC) adjusted by age and sex with representative metabolic markers. Only the CAGs with adjusted *P* (*P*_adj_) < 0.05 are coloured. The category names for CAGs were determined on the basis of the most abundant metabolites in the CAGs. Further details are provided in Supplementary Tables 3–8. FBG, fasting blood glucose; neg., negative; pos., positive. The lipid abbreviations are defined in Supplementary Table 27. **c**, pSC between HOMA-IR and faecal levels of monosaccharides. The coefficients (pSC) and *P*_adj_ values are described (*n* = 282). **d**, Faecal levels of monosaccharides in MetS (*n* = 306). For **a**, the box plots indicate the median (centre line), upper and lower quartiles (box limits), and upper and lower extremes except for outliers (whiskers). conc., concentration. For **c**, the density plots indicate median and distribution. For **a** and **d**, statistical analysis was performed using Kruskal–Wallis tests followed by Dunn’s test (**a**) and rank-based linear regression adjusted by age and sex (**d**); *P < 0.05, **P < 0.01, ***P < 0.001. See the Source Data (**a**) and Supplementary Table 5 (**d**) for exact *P* values. Source Data

To examine how omics data of faecal samples can predict IR, we first compared the area under the curve (AUC) of receiver operating characteristic (ROC) curves on the basis of random-forest classifiers. Predictor variables for the models were selected using the minimum-redundancy maximum-relevance algorithm^15^ from the faecal 16S, metabolome, metagenome and their merged datasets (Supplementary Table 2). We found that the selected features of faecal metabolomic data generally outperformed those of 16S and metagenomics in predicting IR (Fig. 1a), suggesting that faecal metabolomics could be used to study IR pathogenesis.

## Faecal carbohydrates are increased in IR

**Extended Data Fig. 2:**
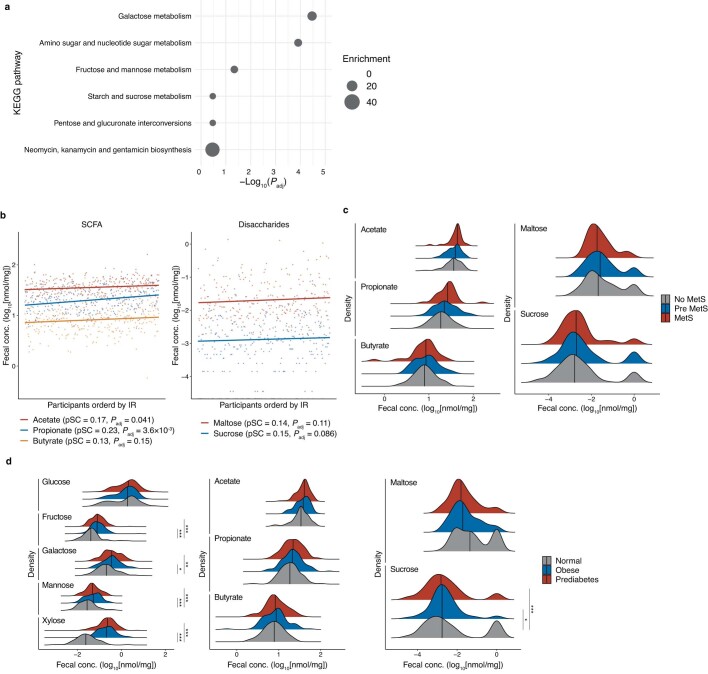
Faecal carbohydrate metabolites are increased in IR and MetS. **a**, The KEGG pathway enrichment analysis of the metabolites in hydrophilic CAGs 5, 8, 12, 15, and 18, which were associated with IR in Fig. 1b. The size of disks shows the enrichment (i.e., the ratio of observed numbers and expected numbers of metabolites in each KEGG pathway). The pathways with raw *P* values < 0.05 are shown in the figure. **b**, Partial correlations between HOMA-IR and faecal levels of short-chain fatty acids (SCFA) such as acetate, propionate, and butyrate (left panel), and disaccharides such as maltose and sucrose (right panel). The coefficients (pSC) and *P* values of partial Spearman’s correlations adjusted by age and sex are described (n = 282). **c**, Faecal levels of SCFA (left panel) and disaccharides (right panel) were compared between no MetS, pre MetS, and MetS (n = 306). **d**, Faecal levels of monosaccharides (left panel), SCFA (middle panel), and disaccharides (right panel) were compared between healthy, obese, and prediabetes (n = 306). Density plots indicate median and distribution. *P_adj_ < 0.05, **P_adj_ < 0.01, ***P_adj_ < 0.001; hypergeometric test with multiple test corrections (**a**) and rank-based linear regression adjusted by age and sex (**c**, **d**). The detailed statistics are reported in Supplementary Table 5, 6.

**Extended Data Fig. 3:**
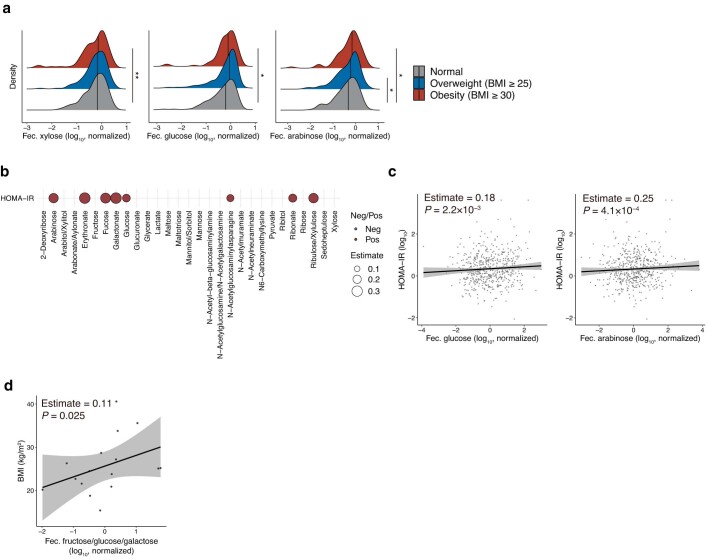
Faecal carbohydrate metabolites are associated with IR-related pathologies. **a**, The faecal xylose, glucose, and arabinose were compared between individuals with normal weight, overweight, and obesity in the TwinsUK cohort (n = 786). **b**, The associations between faecal carbohydrates observed in at least 50% samples and HOMA-IR in the TwinsUK cohort (n = 550). The size and colour of the disks represent the estimate and the direction of the associations. Metabolites with *P*_adj_ < 0.05 are depicted (n = 550). **c**, The associations between faecal glucose and arabinose and HOMA-IR as analysed in Fig. b. The lines and grey zones show the fitted linear regression lines with 95% confidence intervals. The estimates of metabolites and their *P* values are described. **d**, The association between faecal fructose/glucose/galactose and BMI in non-IBD individuals aged > 10 years old in the HMP2 cohort (n = 16). The data were analysed with a generalized linear mixed-effect model with consent age and sex as fixed effects, and the sample collection site as a random effect. The line and grey zone show the fitted linear regression lines with a 95% confidence interval. The estimate and *P* value are described. The first faecal sampling for metabolomics was used to avoid redundancy. Density plots indicate median and distribution. *P < 0.05, **P < 0.01; rank-based linear regression adjusted by age, sex, and zygosity (**a**) and generalized linear mixed-effect models with age, sex, zygosity, and BMI as fixed effects, and sample collection year as a random effect (**b**). The detailed statistics are reported in Supplementary Table 9.

We next searched for the associations between clinical phenotypes and faecal metabolite CAGs (Fig. 1b and Supplementary Tables 3–8). Major confounding factors, namely sex and age, were adjusted throughout the correlation and regression analyses with clinical markers. Among the hydrophilic metabolites, most of the CAGs showing significant associations with IR were those of carbohydrate metabolites, mainly monosaccharides (hydrophilic CAGs 5, 12 and 15; Fig. 1b, top). Short-chain fatty acids (SCFAs), which are known as carbohydrate fermentation products, were also increased in IR (hydrophilic CAG 8). Hydrophilic CAG 18 remained unannotated as it included metabolites from different pathways (Supplementary Table 5). KEGG pathway enrichment analysis of the metabolites in these IR-related hydrophilic CAGs revealed that these metabolites were indeed involved in carbohydrate metabolism (Extended Data Fig. 2a). Specifically, we found that the major monosaccharides such as fructose, galactose, mannose and xylose significantly correlated with IR (Fig. 1c). Among the SCFAs, propionate was particularly increased in IR (Extended Data Fig. 2b), consistent with its role in gluconeogenesis^16^. Faecal monosaccharides were similarly increased in MetS, obesity and prediabetes (Fig. 1d and Extended Data Fig. 2c,d). By contrast, disaccharides showed weak or no association (Extended Data Fig. 2b–d). These findings show that the end products of carbohydrate degradation—such as monosaccharides, which are readily absorbed and used by the host—are particularly increased in the faeces of individuals with IR and MetS. Supporting these findings, our analysis of previously published faecal metabolomics data from the TwinsUK cohort^17^ showed that faecal monosaccharides, notably glucose and arabinose, were positively associated with obesity and HOMA-IR, both of which relate to IR (Extended Data Fig. 3a–c and Supplementary Table 9). Similarly, the peak intensity of faecal fructose, glucose and galactose was associated with BMI in a small number of individuals without inflammatory bowel disease (IBD) from HMP2 data^18^ (Extended Data Fig. 3d). Together, these findings indicate that faecal carbohydrates are increased in IR and related pathologies and that this alteration is consistently observed across populations.

**Extended Data Fig. 4:**
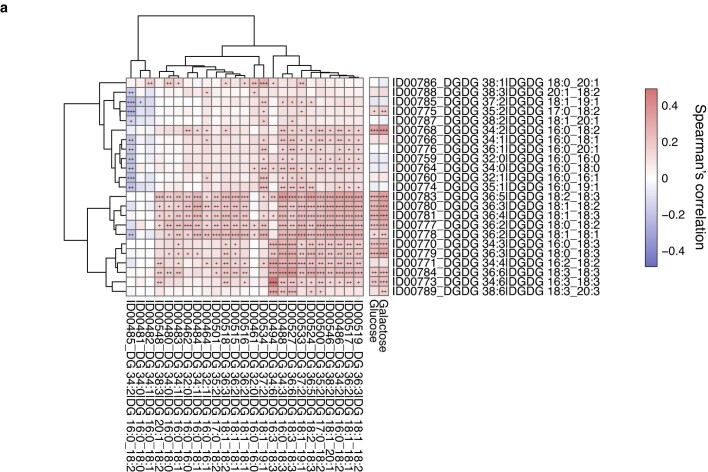
Faecal DGDG and their precursors. **a**, The associations between the faecal levels of digalactosyl/glucosyldiacylglycerols (DGDGs) in lipid CAG 11 from Fig. 1b, and their precursor DGs (left panel) and monosaccharides, i.e., glucose and galactose (right panel) (n = 282).

In addition to hydrophilic metabolites, faecal lipid CAGs were also associated with IR (Fig. 1b). Lysophospholipids, bile acids and acylcarnitine were associated with IR and MetS as reported previously^19^. Among them, a lipid CAG largely consisting of digalactosyl/glucosyldiacylglycerol (DGDG) (lipid CAG 11) came to our attention as DGDG is reportedly derived from bacteria^20,21^. These lipids contain glucose and/or galactose in their structures, although their biological functions in mammals are largely unclear. Most of the DGDGs in this cluster showed positive correlations with some of the precursor diacylglycerols and monosaccharides (that is, glucose and galactose) (Extended Data Fig. 4a). As diacylglycerols are deeply involved in IR pathogenesis^22^, the biological functions of this metabolite class are of particular interest. Notably, DGDGs with different acyl chains in lipid CAG 41 showed no association with IR (Supplementary Table 7), implying that the differences in acyl chains of lipids may have a physiological importance as reported previously^23^.

## Microorganism–metabolite relationships in IR

**Fig. 2:**
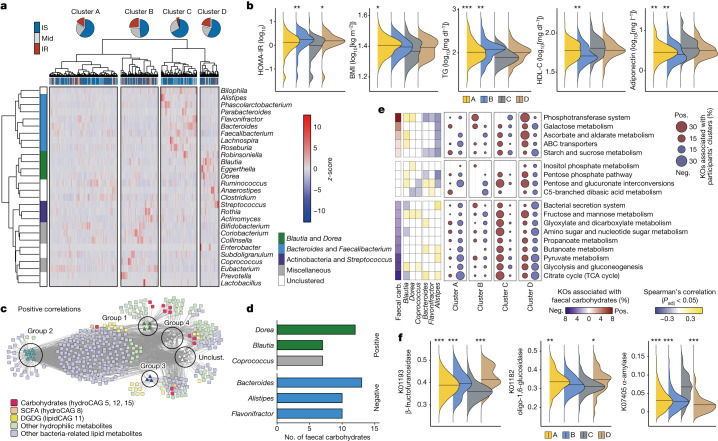
IR-associated faecal metabolites are associated with altered gut microbiota and microbial genetic functions. **a**, Co-abundance clusters of bacteria at the genus level and their abundance (*n* = 282). The participants were classified into four clusters, A to D, according to their taxonomic profiles. The proportion of individuals with IR are shown. Mid, intermediate. **b**, HOMA-IR, BMI, triglycerides (TG) and HDL-C levels among the participant clusters. **c**, Bacteria–metabolite networks of co-abundance microbial groups from **a** and faecal metabolites (*n* = 282). All faecal hydrophilic and bacteria-related lipid metabolites were included. Only interactions with positive and significant (*P*_adj_ < 0.05) Spearman’s correlations are shown. The metabolites in CAGs relating to carbohydrates in Fig. 1b are highlighted in red. Unclust., unclustered. **d**, The number of significant positive and negative correlations between genera and faecal carbohydrates. The top five genera in each correlation are shown. **e**, KEGG pathways relating to carbohydrate metabolism and membrane transport, faecal carbohydrates, the top three genera positively or negatively correlated with faecal carbohydrates, and the participant clusters. KEGG orthologues significantly (*P*_adj_ < 0.05) associated with the metabolite (left) and taxonomic abundance (right) are summarized as the percentage enrichment among KEGG pathways. The median percentage of 15 faecal carbohydrates (carb.) is shown in colour (blue to red) on the left, whereas the percentage enrichment is shown as the disk size on the right; the Spearman’s correlations between pathway-level abundance and six genera are shown in colour (blue to yellow) in the middle (*n* = 266). **f**, The abundance of representative KEGG orthologues involved in glycosidase among the participant clusters (*n* = 266). The abundance was transformed by arcsine square root transformation. The density plots in **b** and **f** indicate the median and distribution. Statistical analysis was performed using rank-based linear regression adjusted by age and sex (**b**; Supplementary Table 10), two-sided Wilcoxon rank-sum tests with multiple-testing correction (**e**; Supplementary Table 16), and Kruskal–Wallis tests with Dunn’s test (**f**; Supplementary Table 18). *P < 0.05, **P < 0.01, ***P < 0.001 in comparison to cluster C (with the lowest proportion of IR) (**b** and **f**).

**Extended Data Fig. 5:**
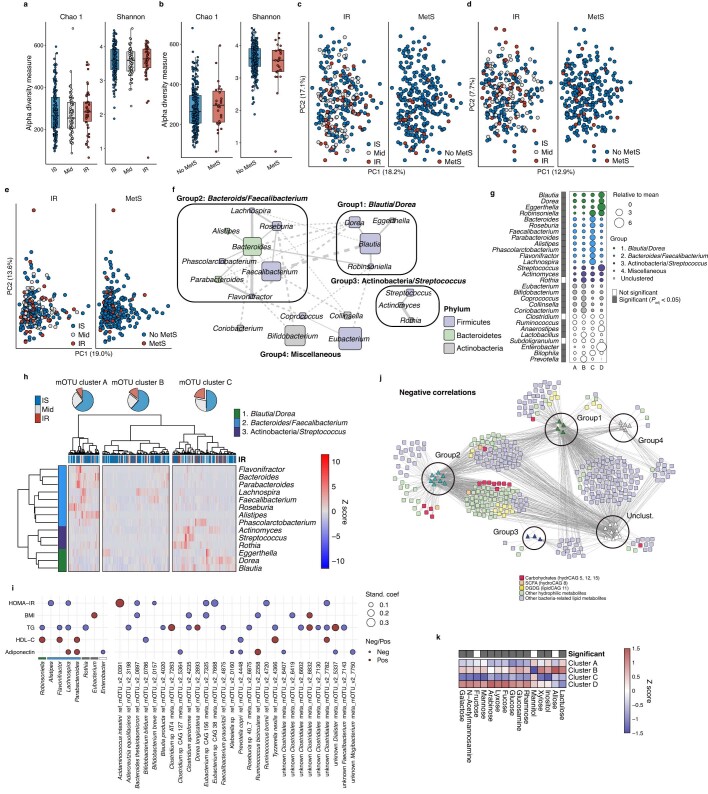
Faecal microbiota in IR. **a**, **b**, Chao1 and Shannon’s alpha diversity indices in IR and MetS (n = 282). **c**, **d**, PCoA plots of Bray-Curtis dissimilarity, showing the variations of faecal microbiota at the genus level based on 16S rRNA gene sequencing (**c**), and at the species (mOTU) level based on shotgun sequencing (**d**), clustered by IR or MetS (n= 282). Dots represent individual data summarized into PCo1 and PCo2. **e**, PCA plots showing the variations of KEGG orthologues based on shotgun metagenomic sequencing clustered by IR or MetS (n = 266). Dots represent individual data summarized into PC1 and PC2. **f**, Co-abundance groups of genus-level microbes and their abundance in the participant clusters defined in Fig. 2a. Co-abundance was determined based on compositionality-corrected Spearman’s correlations, with *P*_adj_ < 0.05 considered significant. The disk size represents the median abundance in the participants. Three co-abundance groups were determined based on their networks, while the rest of the microbes were named as “miscellaneous”. **g**, The co-abundance groups of genus-level microbes and their abundance in the participant clusters. Those not clustered by compositionality-corrected Spearman’s correlations in **f** were shown as “Unclustered”. The size of the disks represents overabundance to the mean in four clusters of participants determined in Fig. 2a. The far-left column shows the genera that exhibit significant differences among the four clusters. **h**, The co-abundance clusters of microbes at the genus level using the shotgun metagenomic data and their abundance (n = 266). The genera forming distinct groups in **f**, i.e., groups 1, 2, and 3, were included in this analysis. The participants were clustered into three mOTU clusters A to C based on the heatmap clustering. The proportion of individuals with IS, intermediate, and IR are shown in the pie charts above the heatmap as Fig. 2a. **i**, The associations between representative metabolic markers and genera (left panel, n = 282) and mOTU (right, n = 266). Only those with significant associations with metabolic markers are depicted. The disk size and colour represent absolute values of standardized coefficient and the direction of associations. The detailed statistics are reported in Supplementary Table 11. **j**, Microbe-metabolite networks of IR- or and IS-associated co-abundance microbial groups from Fig. 2a and faecal metabolites (n = 282). All faecal hydrophilic metabolites and faecal microbe-related lipid metabolites were included in the analysis. Only those with negative Spearman’s correlation between the genus-level microbial abundance and the metabolites with *P*_adj_ < 0.05 are shown, which is complementary to Fig. 2c. The metabolites in CAGs relating to carbohydrates shown in Fig. 1b are highlighted in red. **k**, The relative abundance of IR-associated faecal carbohydrates in the participant clusters. The metabolites significantly different among these four clusters are coloured grey in the top row. **a**, **b**, Box plots indicate the median, upper and lower quartiles, and upper and lower extremes except for outliers. Kruskal-Wallis test (**g**, **k**). See the Source Data (**g**) for exact *P* values. Source Data

We next investigated the alteration in gut microbiota and the functions of gut microbiota that are associated with IR. Gut microbiota diversity varied among individuals (Extended Data Fig. 5a–e). We then profiled the genus-level microbial composition of the study participants using 16S rRNA sequencing data^24^ and identified four bacterial groups (Extended Data Fig. 5f). Group 1 was dominated by the Lachnospiraceae family such as *Blautia* and *Dorea*, whereas group 2 was characterized by Bacteroidales (such as *Bacteroides*, *Parabacteroides* and *Alistipes*) and *Faecalibacterium*. Group 3 contained Actinobacteria genera. Group 4 did not form a distinct network. We could further classify the study participants into four clusters, A to D, on the basis of their taxonomic profiles (Fig. 2a). Individuals in cluster C distinctly harboured group 2 with Bacteroidales, whereas those in cluster D showed a higher abundance of group 1 and 3 bacteria (Extended Data Fig. 5g). Notably, the proportion of IR (Fig. 2a; *P* = 0.0071) was significantly lower in cluster C. Other metabolic parameters associated with IR and MetS such as HOMA-IR, BMI, triglycerides, HDL-cholesterol (HDL-C) and adiponectin were also different between cluster C (with the lowest proportion of IR) and the other three clusters (Fig. 2b and Supplementary Table 10). The proportion of IR among individuals with abundant group 1 and 3 bacteria was consistently higher than those with abundant group 2 bacteria, as identified on the basis of shotgun metagenomics data (Extended Data Fig. 5h). HOMA-IR showed negative associations with the genus *Alistipes* in the Rikenellaceae family and several species from *Bacteroides*, *Bifidobacterium* and *Ruminococcus* (Extended Data Fig. 5i and Supplementary Tables 11 and 12), partly recapitulating previous reports regarding individuals with obesity^25–27^. Notably, different genera and species correlated with other clinical markers, suggesting that the individual association between microbial taxa and clinical manifestation is not as robust as in the co-abundance analysis.

We next constructed a microorganism–metabolite network on the basis of the significant positive or negative correlations (Supplementary Table 13). Although faecal SCFAs and lipids such as DGDG correlated with both IR- and IS-associated bacterial groups, IR-associated faecal carbohydrates predominantly correlated with genera in groups 1 and 4, the most prominent being *Dorea* in Lachnospiraceae (Fig. 2c,d). By contrast, the majority of these carbohydrates negatively correlated with IS-associated genera in group 2 bacteria such as *Bacteroides*, *Alistipes* and *Flavonifractor* (Fig. 2d and Extended Data Fig. 5j), with minimal correlations with bacteria in group 1. Accordingly, the faecal carbohydrate levels were distinctly different among the participant clusters (Extended Data Fig. 5k). Previous studies have suggested that several Lachnospiraceae species are involved in polysaccharide fermentation^28,29^, while *Alistipes* is increased on an animal-based diet rather than a polysaccharide-rich diet^30^. These findings highlight a tight connection between carbohydrate-degradation products and IR- and IS-associated bacteria, suggesting that these bacteria may be involved in the aberrant faecal carbohydrate profile in IR.

**Extended Data Fig. 6:**
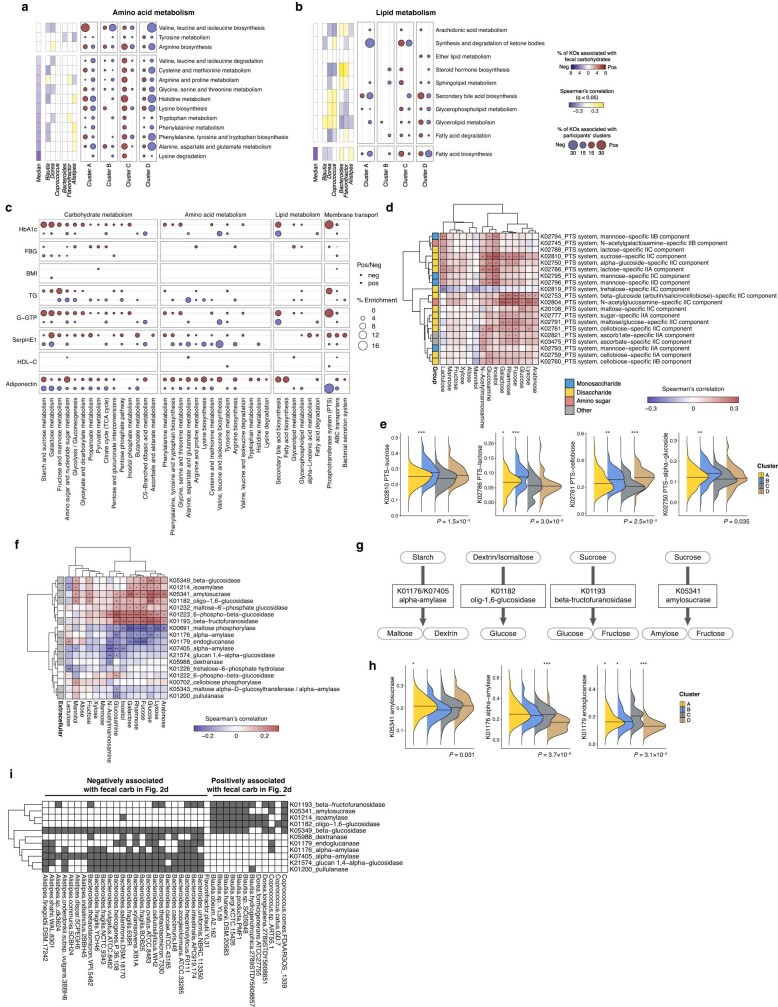
Microbial carbohydrate metabolism is altered in IR. **a**, **b**, The associations between the KEGG pathways relating to amino acid metabolism (**a**) and lipid metabolism (**b**), faecal carbohydrates, top three genera positively or negatively correlated with faecal carbohydrates in Fig. 2d, and the participant clusters defined in Fig. 2a. KEGG orthologues significantly (*P*_adj_ < 0.05) associated with the metabolite (left) and taxonomic abundance (right) are summarized as percent enrichment among the KEGG pathways. The median % of 15 faecal carbohydrates are coloured in the left panel whereas % enrichment is depicted as the disk size in the right panel. The Spearman’s correlations between pathway-level abundance and 6 genera were analysed in the middle panel (n = 266). **c**, The associations between representative metabolic markers and the KEGG pathways relating to carbohydrate metabolism, amino acid metabolism, lipid metabolism, and membrane transport defined in the KEGG orthology database. The pathways with significant associations with metabolic markers are included in the plots. The disk size and colour represent % enrichment and the direction of associations, and only significant (*P*_adj_ < 0.05) associations are depicted (n = 266). **d**, Spearman’s correlation between KEGG orthologues associated with phosphotransferase system (PTS) and faecal carbohydrate metabolites. KEGG orthologues significantly (*P*_adj_ < 0.05) associated with faecal metabolites are coloured red or blue (n = 266). The far-left column shows the type of carbohydrate metabolites that each PTS gene is involved in. **e**, The abundance of representative KEGG orthologues involved in PTS were compared among four participant clusters (n = 266). The abundance was transformed by arcsine square root transformation. **f**, Spearman’s correlation between KEGG orthologues significantly associated with glycoside hydrolases in starch and sucrose metabolism (KEGG pathway #00500) and faecal carbohydrate metabolites (n = 266). The far-left column shows whether the genes were predicted to function as extracellular enzymes. **g**, Representative pathways in starch and sucrose metabolism (KEGG pathway #00500) relating to glycosidase activities to degrade poly- and oligosaccharides into monosaccharides. **h**, The abundance of representative KEGG orthologues involved in glycosidase were compared among four participant clusters (n = 266). The abundance was transformed by arcsine square root transformation. **i**, The presence and absence of KEGG orthologues predicted to function as extracellular enzymes in 45 strains. The strains from the top three genera positively or negatively correlated with faecal carbohydrates shown in Fig. 2d, i.e., *Bacteroides*, *Alistipes*, *Flavonifractor*, *Dorea*, *Blautia*, and *Coprococcus*, were included in this analysis. Density plots indicate median and distribution (**e**, **h**). *P < 0.05, **P < 0.01, ***P < 0.001 in comparison to cluster C (with the lowest proportion of IR); Kruskal-Wallis test with Dunn’s test (**e**, **h**) (Supplementary Table 18).

The IR-associated faecal carbohydrates were also correlated with KEGG pathways relating to carbohydrate metabolism and transportation, such as the phosphotransferase system (PTS), starch and sucrose metabolism, and galactose metabolism, while negatively associated with pathways relating to carbohydrate catabolism, such as glycolysis and pyruvate metabolism (Fig. 2e and Supplementary Tables 14 and 15). These pathways were also distinctly correlated with the participant clusters defined in Fig. 2a and the genera relating to carbohydrates defined in Fig. 2d. Amino acid metabolism was also different, particularly between clusters C and D, whereas lipid metabolism did not show distinct associations with microbiota (Extended Data Fig. 6a,b and Supplementary Table 16). Although carbohydrate pathways such as PTS and starch and sucrose metabolism showed strong positive associations with HbA1c and γ-GTP, the associations with other IR markers were generally sparse (Extended Data Fig. 6c and Supplementary Table 17), suggesting that metabolites are more sensitive to the clinical manifestations as shown in Fig. 1a. PTS is an essential component for bacteria to incorporate sugars into themselves as energy sources^31^. Detailed analyses of KEGG orthologues revealed that faecal carbohydrates and participant clusters mainly correlated with PTSs relating to disaccharides and amino sugars (Extended Data Fig. 6d,e and Supplementary Table 18), suggesting that the preference of sugar use by microbiota through PTS may affect the metabolite levels. Glycosidases, which catalyse the breakdown of oligo- and disaccharides^32^, were also associated with faecal monosaccharides (Extended Data Fig. 6f). Extracellular glucosidases such as β-fructofuranosidase (K01193, KEGG Orthology database), amylosucrase (K05341, KEGG Orthology database) and oligo-1,6-glucosidase (K01182, KEGG Orthology database), which were predicted to degrade sucrose and dextrin into glucose and fructose (Extended Data Fig. 6g,h), showed the highest positive correlations, especially with faecal glucose. By contrast, glucosidases relating to starch use such as α-amylases (K01176 and K07405, KEGG Orthology database) were negatively linked with faecal carbohydrates. Importantly, the abundance of these glycosidase genes was significantly different between participant cluster C and the other three clusters, suggesting that taxonomic profiles largely explain the variations of glucosidases (Fig. 2f, Extended Data Fig. 6h and Supplementary Table 18). Consistently, disaccharide-breakdown genes were predominantly conserved in the genomes of *Blautia* and *Dorea* abundant in cluster D, whereas they were almost lacking in Bacteroidales abundant in cluster C (Extended Data Fig. 6i). Together, our findings reveal four distinct populations with unique taxonomic profiles and carbohydrate metabolisms characterized by sugar use and degradation, which correlate with IR and its related markers.

## Faecal carbohydrates and inflammation in IR

**Fig. 3:**
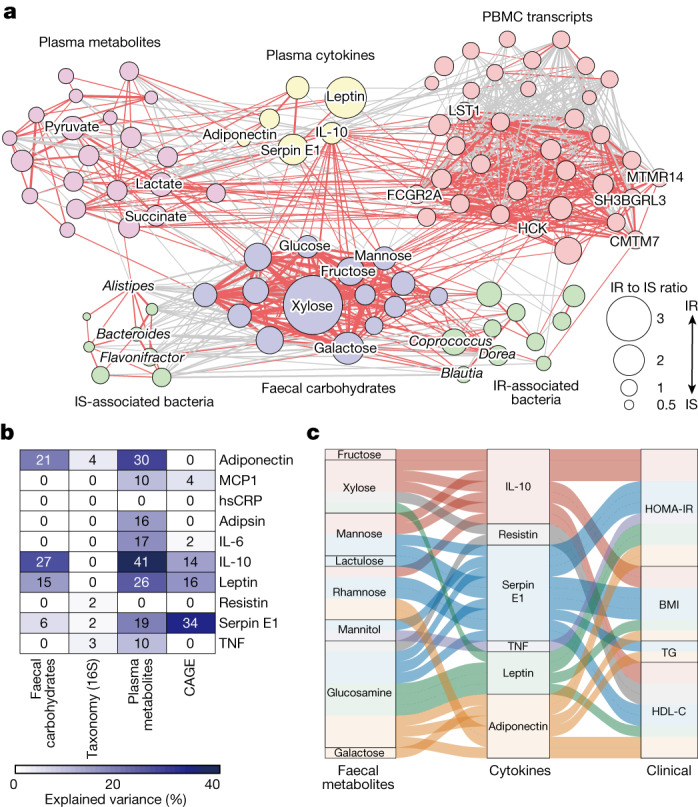
Faecal carbohydrate metabolites are associated with cytokine levels in IR. **a**, The networks between faecal carbohydrate metabolites (purple), faecal bacteria (green), plasma hydrophilic metabolites (pink), cytokines (yellow) and PBMC genes (red) constructed on the basis of the IS, intermediate (that is, HOMA-IR >1.6 and <2.5) and IR samples available for all omics information (*n* = 46, 70 and 275). Host-derived markers significantly associated with IR (Supplementary Tables 19–21), 15 faecal carbohydrates and 20 genera identified in Fig. 1b and Extended Data Fig. 5f, respectively, were included in the analysis. To construct the omics network, pairwise pSC adjusted by age, sex, BMI and FBG were calculated, and the interactions with *P*_adj_ < 0.05 are shown. The line widths show the absolute values of coefficients, and the red and grey lines show positive and negative correlations, respectively. The disk sizes show the ratio of median abundance in IR over IS (*n* = 46 and 157). Detailed information with complete annotations is shown in Extended Data Fig. 7c and Supplementary Table 22. **b**, The explained variance of ten plasma cytokines predicted by each omics dataset using random-forest classifiers. **c**, An alluvial plot showing the plasma cytokines significantly mediated the in silico effects of faecal carbohydrates on host metabolic markers. The lines show the mediation effects and the colours represent the associations mediated by individual cytokines. Details are provided in Supplementary Table 23.

**Extended Data Fig. 7:**
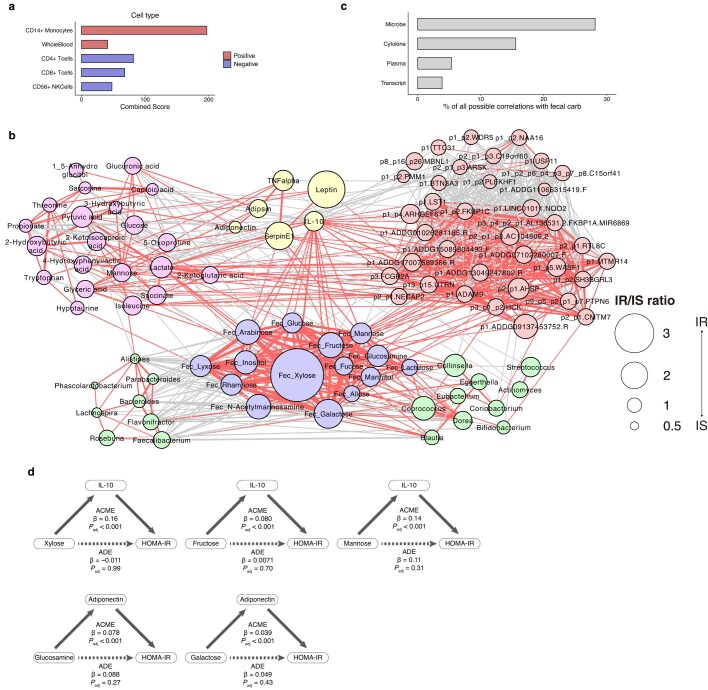
Cytokine and faecal metabolite interactions in IR. **a**, Cell-type gene set enrichment analysis based on the Human Gene Atlas database using Enrichr. Annotated peripheral blood mononuclear cell (PBMC) transcripts positively or negatively associated with IR (Supplementary Table 21) were analysed (n = 275). Red and blue colour scales represent IR and IS-associated cell types, respectively (please refer to Methods for details). **b**, The cross-omics network shown in Fig. 3a with the annotations. **c**, The number of correlations between faecal carbohydrates and other omics elements shown in Fig. 3a. The proportion to all possible correlations is shown. **d**, Representative causal mediation models analysing the effects of IL-10 and adiponectin mediating *in silico* relationships between faecal carbohydrates and HOMA-IR. Causal mediation analysis with multiple test corrections were used to test significance. Estimates (β) and *P*_adj_ values of average causal mediation effects (ACME), which are the indirect effects between the metabolites and host markers mediated by cytokines, and average direct effects (ADE), which are the direct effects controlling for cytokines, are described. Age and sex were adjusted in the models. The detailed information is reported in Supplementary Table 23.

Consistent with previous reports^1,2^, the host cytokine, metabolomic and transcriptomic signatures were highly associated with IR (Supplementary Tables 19–21). Moreover, many of these PBMC genes were functionally involved in inflammation (Extended Data Fig. 7a) and possibly derived from monocytes (Supplementary Table 21). Several studies have suggested that microbial components such as lipopolysaccharides have a role in facilitating inflammation of metabolic diseases^33,34^. However, it remains unclear whether microbial metabolism is involved in low-grade inflammation. We therefore tried to infer possible associations between host inflammatory signatures of IR and faecal carbohydrates. First, the cross-omics correlation-based network with individual metabolites, bacteria, transcripts and cytokines associated with IR revealed that faecal carbohydrates were strongly tied with both bacteria and host IR-related signatures, especially cytokines, suggesting that these metabolites are the hubs of the host–microorganism network in IR (Fig. 3a, Extended Data Fig. 7b,c and Supplementary Table 22). Differential abundance, calculated as the ratio of their abundance in IR and IS, was most pronounced in the associations between faecal carbohydrates and cytokines. Notably, IL-10, a plasma cytokine, showed the most prominent associations with faecal carbohydrates and modestly with PBMC-derived transcripts, supporting recent studies showing its paradoxical effect to facilitate IR^35–37^. Faecal carbohydrates moderately explained the variance of IL-10 and, to a lesser extent, adiponectin, leptin and serpin E1, suggesting that faecal carbohydrates are particularly associated with these cytokines (Fig. 3b). Although the proportions of variance explained by faecal carbohydrates were lower than by plasma metabolites, they were much higher than those by genus-level abundance, highlighting the role of faecal metabolites linking gut microbiota and host inflammatory responses. We next sought to infer whether these cytokines mediated the effects of faecal carbohydrates on host metabolism using causal mediation analyses^38^. We found that IL-10, serpin E1, adiponectin and leptin mediated most in silico causal relationships between faecal carbohydrates and host IR markers such as HOMA-IR (Fig. 3c, Extended Data Fig. 7d and Supplementary Table 23). Notably, there were unique correspondences between metabolites and cytokines; for example, IL-10 mediated the effects of fructose, mannose, xylose and rhamnose, but not other metabolites. Although the biological importance of these unique correspondences remains to be investigated, the combined analyses of faecal microbiota, metabolome and host inflammatory phenotypes in IR suggest a previously unrecognized interaction, whereby excessive monosaccharides may affect host cytokine expression.

## IS-associated bacteria in experimental models

**Extended Data Fig. 8:**
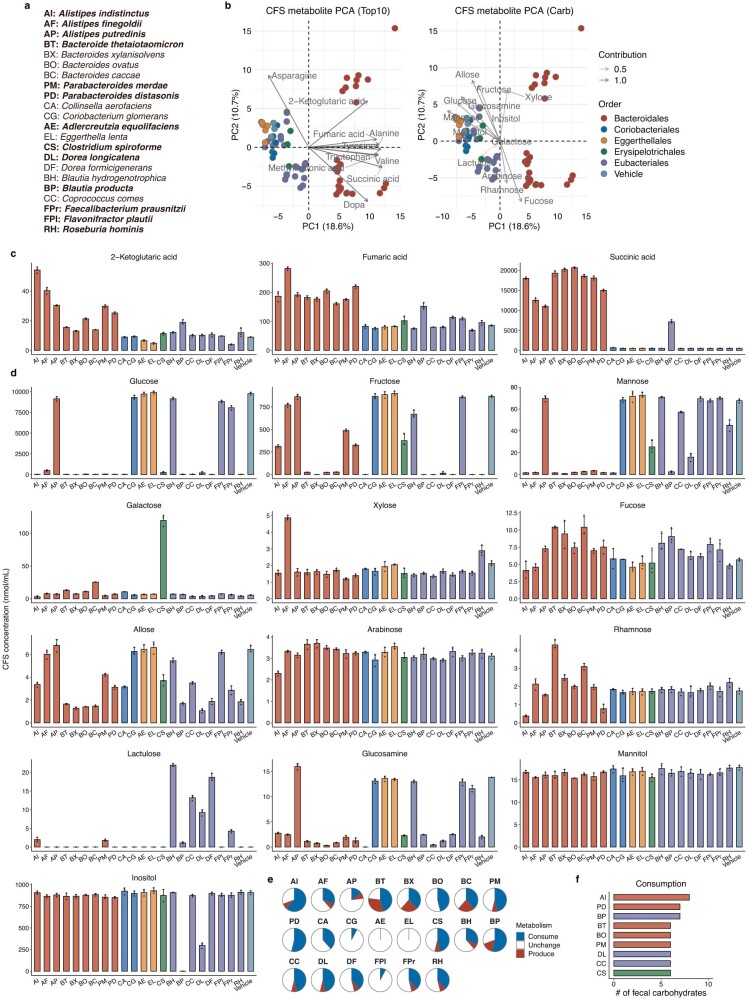
Bacteroidales strains distinctly alter metabolites in the culture supernatant. **a**, **b**, PCA plots of metabolites in cell-free supernatants of 22 bacterial strains listed in (**a**). These strains were selected based on the findings from the genus-level co-occurrence (Fig. 2a, b) and the species-level profiles (Extended Data Fig. 5i). The strains from genera and species relating to IR-related markers shown in Extended Data Fig. 5i are particularly highlighted in boldface. The top 10 metabolites contributing to the PCA separation (left panel) and 13 out of 15 IR-related carbohydrates identified in Fig. 1b (right panel) are biplotted on the PCA plot, respectively (**b**). **c**, **d**, The levels of carbohydrate fermentation products (**c**) and carbohydrates relating to IR in the human cohort (**d**) in the cell-free supernatants. **e**, Pie charts summarizing the consumption and production of carbohydrates shown in (**d**). Those significantly decreased or increased compared with the vehicle control group were considered as consumption or production. **f**, The top consumers of carbohydrates, which summarizes the results shown in (**e**). Representative data of two independent experiments. **c**, **d**, Data are mean and s.d. The detailed statistics are reported in Supplementary Table 24 (n = 3 per group).

The above findings from human multi-omics analyses revealed an association between carbohydrate metabolites and IR pathology. To address the causal relationship between gut microbiota, faecal carbohydrates and metabolic diseases, we first analysed metabolites in the bacterial culture of 22 human faecal IS- and IR-associated bacteria. These bacteria were selected on the basis of the findings from the genus-level co-occurrence (Fig. 2a,b) and the species-level (Extended Data Fig. 5i) profiles. Principal component analysis plots of 198 metabolites indicated that Bacteroidales, a representative IS-associated bacterial order, showed a distinct metabolic profile along PC1 (Extended Data Fig. 8a,b and Supplementary Table 24). The top 10 metabolites contributing to the group separation included several amino acids and fermentation products such as succinate and fumarate, and the majority of these metabolites were preferentially produced by Bacteroidales (Extended Data Fig. 8b,c). We detected 13 out of 15 carbohydrates associated with IR (Fig. 1b) in the bacterial culture (Extended Data Fig. 8b). Most of these carbohydrates were plotted negatively along PC1, suggesting that these metabolites were negatively associated with Bacteroidales. Glucose, mannose and glucosamine were preferentially consumed by Bacteroidales compared with the other orders, whereas lactulose was mainly produced by *Eubacteriales* (Extended Data Fig. 8d). *Alistipes indistinctus* was the most potent in consuming a wide variety of carbohydrates (Extended Data Fig. 8e,f). These findings show that Bacteroidales species are potent consumers of several carbohydrates, driving the production of their fermentation products.

**Fig. 4:**
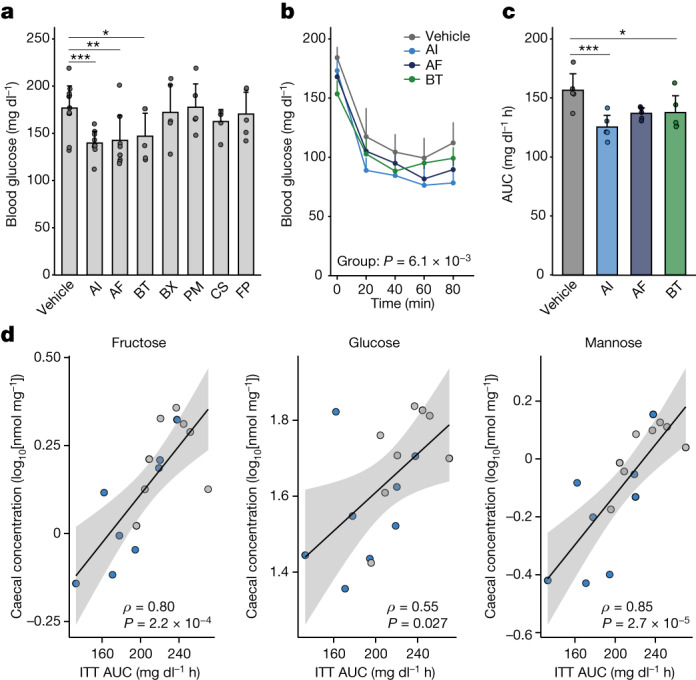
IS-associated bacteria ameliorate IR in experimental models. **a**, Postprandial blood glucose in mice fed a high-fat diet at 4 weeks after the initiation of bacterial administration. The abbreviations are defined in Extended Data Fig. 8a. *n* = 12 (vehicle), *n* = 10 (*A. indistinctus* and *A. finegoldii*) and *n* = 5 (other groups) mice. **b**,**c**, Blood glucose levels during the insulin tolerance test (**b**) and the AUC (**c**) (*n* = 5 per group). **d**, The correlations between the AUC of the insulin tolerance test and caecal levels of fructose, glucose and mannose in the *A. indistinctus* (sky blue) or vehicle (grey) groups. Spearman’s coefficients (*ρ*) and *P* values are shown. The lines and grey zones show the fitted linear regression lines with 95% confidence intervals. ITT, insulin tolerance test. Representative data of two (**a** and **d**) or three (**b** and **c**) independent experiments. For **a**–**c**, data are mean ± s.d. Statistical analysis was performed using Kruskal–Wallis tests with Dunn’s test (**a** and **c**) and two-way repeated-measures analysis of variance (ANOVA) (**b**). *P < 0.05, **P < 0.01, ***P < 0.001 (**a** and **c**). Exact *P* values for **a** and **c** are provided in the Source Data. Source Data

**Extended Data Fig. 9:**
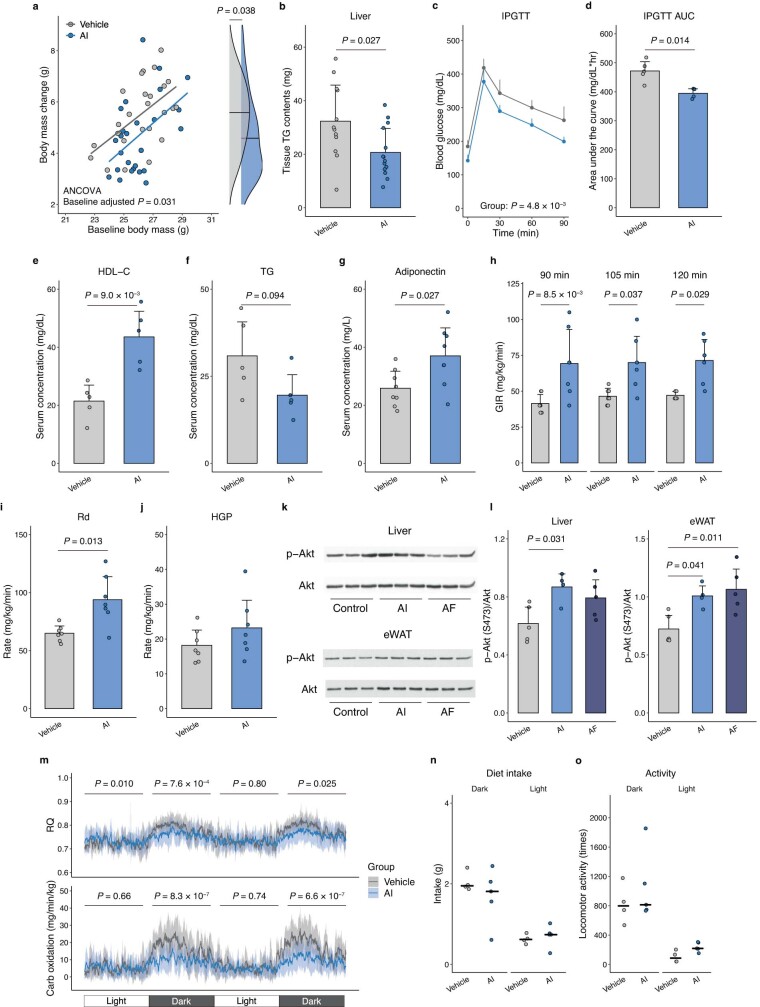
*Alistipes indistinctus* ameliorates IR. **a**, Body mass change from the baseline. The *P* value adjusted by baseline body mass by ANCOVA are shown (n = 25 and 26 for control and *A. indistinctus* (AI) groups, respectively. Pooled data of three independent experiments). **b**, TG contents in the liver (n = 12 and 14 for control and AI groups, respectively. Pooled data of two independent experiments). **c**, **d**, The blood glucose levels (**c**) and AUC (**d**) in intraperitoneal glucose tolerance test (IPGTT) (n = 5 and 4 for control and AI groups, respectively). **e–g**, Serum levels of HDL-cholesterol (HDL-C, **e**), triglycerides (TG, **f**), and adiponectin (**g**) (n = 5 per group in **e** and **f**, n = 8 per group in **g**). **h**, Glucose infusion rate (GIR) during hyperinsulinemic-euglycemic clamp (n = 7 per group). The rates at 90, 105, and 120 min after the start of insulin infusion were shown as representative of steady-state conditions of euglycemia. **i**, **j**, Whole-body glucose disposal rate (Rd, **i**) and hepatic glucose production (HGP, **j**) measured with hyperinsulinemic-euglycemic clamp (n = 7 per group). **k**, **l**, Representative images of phosphorylated Akt (p-Akt) at S473 and total Akt in the liver and epidydimal fat (eWAT) in mice administered *Alistipes indistinctus* (AI), *Alistipes finegoldii* (AF), and PBS as vehicle control (**k**). The protein expression of p-Akt was normalized to that of total Akt (n = 4 vs 5 vs 5) (**l**). The raw images of blotting membranes are shown in Supplementary Fig. 1 (n = 3 per group). **m–o**, Respiratory quotient (RQ) and carbohydrate oxidation rate (**m**), diet intake (**n**), and locomotor activity (**o**) after one-week bacterial administration (n = 4 and 5 for control and AI groups, respectively). *P* values for interactions between time and group are described in (**m**). Other metabolic measures are reported in Supplementary Table 25. Representative data of two independent experiments (**c–g**, **k–o**). **a**, Density plots indicate median and distribution. **b–j**, **l**, **m**, Data are mean and s.d. ANCOVA (main panel) with unadjusted linear regression (right panel) (**a**), two-sided Wilcoxon rank-sum test (**b**, **d–g**, **i, j**), two-way repeated measure ANOVA (**c**), Two-way ANOVA (**h**) and one-way ANOVA (**l**) with Tukey’s test, two-way mixed ANOVA (**m**), and Kruskal-Wallis test (**n**, **o**). Source Data

We next tested the potential therapeutic effects of seven candidate bacteria shown to be associated with IS in human cohort findings. Postprandial blood glucose levels were particularly reduced in mice administered with *A. indistinctus*, *Alistipes finegoldii* and *Bacteroides thetaiotaomicron* that were fed a high-fat diet (Fig. 4a). Insulin tolerance tests also revealed that these strains ameliorated IR, most prominently by *A. indistinctus* administration (Fig. 4b,c). *A. indistinctus* administration ameliorated body mass gain, ectopic triglyceride accumulation in the liver and glucose intolerance (Extended Data Fig. 9a–d). Serum levels of HDL-C, adiponectin and, to a lesser extent, triglycerides, were also improved in mice that were treated with *A. indistinctus* (Extended Data Fig. 9e–g). The findings of the hyperinsulinaemic–euglycaemic clamp analysis indicated that *A. indistinctus* administration significantly improved IR and, particularly, whole-body glucose disposal (Extended Data Fig. 9h–j). Phosphorylation of AKT in the liver and epididymal fat was increased in mice treated with *A. indistinctus* and *A. finegoldii* mice (Extended Data Fig. 9k,l), suggesting that insulin signalling was improved in the liver and adipose tissue. These findings reveal a potency of *A. indistinctus* administration in ameliorating diet-induced obesity and IR.

**Extended Data Fig. 10:**
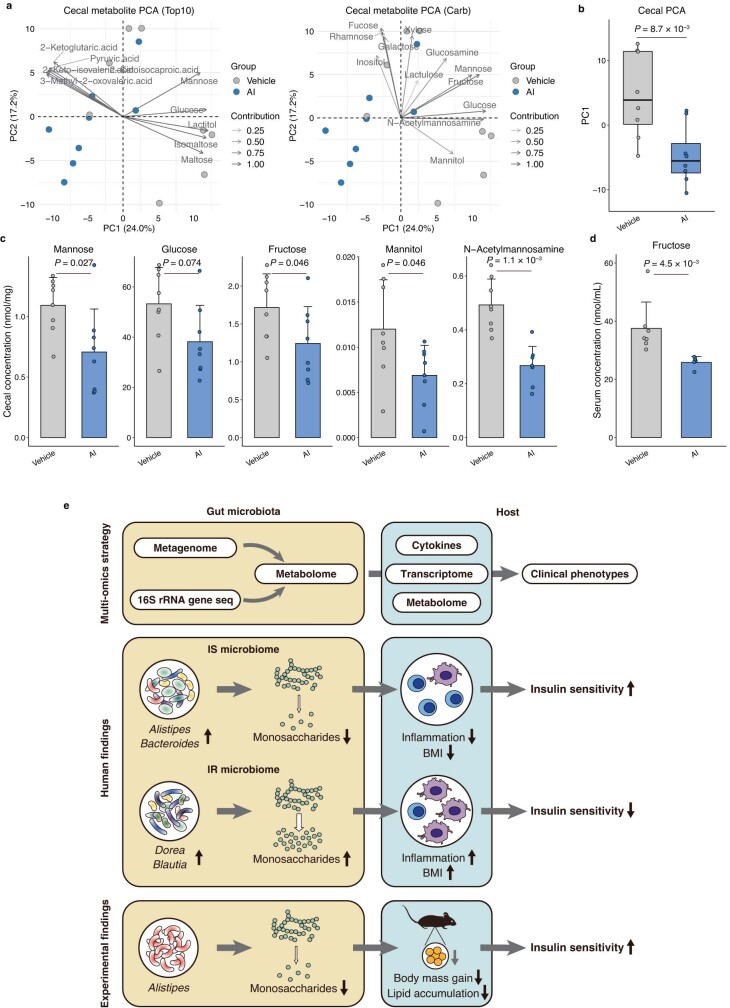
*Alistipes indistinctus* reduces intestinal carbohydrates. **a**, PCA plots of metabolites in caecal contents of AI-administered mice. The top 10 metabolites contributing to the PCA separation (left panel) and 12 out of 15 IR-related carbohydrates identified in Fig. 1b (right panel) are biplotted on the PCA plot, respectively (n = 8 per group). **b**, The PC1 of PCA plots in Fig. a (n = 8 per group). **c**, Caecal levels of representative IR-related carbohydrates observed in AI-administered mice (n = 8 per group). The detailed statistics of all caecal metabolites are reported in Supplementary Table 26. **d**, Serum levels of fructose in AI-administered mice (n = 7 and 5 for control and AI groups, respectively). **e**, A schematic summary. In this study, we combined faecal metabolome, 16S rRNA gene sequencing, and metagenome data with host metabolome, transcriptome, and cytokine data to comprehensively delineate the involvement of gut microbiota in IR (upper panel). Carbohydrate degradation products such as monosaccharides are prominently increased in IR (middle panel). Metagenomic findings show that the degradation and utilization of poly- and disaccharides are facilitated in IR and that these microbial functions are strongly associated with faecal monosaccharides. Further analysis also suggests that the effects of these metabolites on host metabolic parameters such as BMI are in part mediated by specific cytokines. Finally, our animal experiments provide evidence showing that oral administration of AI, a candidate strain selected based on human cohort findings, reduces intestinal carbohydrates and lipid accumulation, thereby leading to the amelioration of IR (lower panel). Taken together, our study provides novel insights into the mechanisms of host-microbe interplays in IR. Representative data of two independent experiments. **b**, Box plots indicate the median, upper and lower quartiles, and upper and lower extremes except for outliers. **c**, **d**, Data are mean and s.d. Two-sided Wilcoxon rank-sum test (**b–d**). Source Data

Mechanistically, metabolic measurement revealed that carbohydrate oxidation was significantly reduced in mice that were treated with *A. indistinctus*, implying that carbohydrate use is limited (Extended Data Fig. 9m and Supplementary Table 25). As dietary intake and locomotor activity remained unchanged (Extended Data Fig. 9n,o), we reasoned that host-accessible carbohydrates in the intestine were reduced by treatment with *A. indistinctus*. In this regard, *A. indistinctus* administration substantially altered caecal metabolites, characterized by a reduction in several carbohydrates including fructose, a lipogenic monosaccharide^39^ (Extended Data Fig. 10a–c and Supplementary Table 26). Fructose was similarly reduced in the serum (Extended Data Fig. 10d). Importantly, the AUC of insulin tolerance test was positively correlated with the caecal monosaccharides fructose, glucose and mannose (Fig. 4d). Collectively, these findings reveal that *A. indistinctus* ameliorates IR and affects intestinal carbohydrate metabolites in mice, supporting our observations in the human cohort.

## Discussion

To deepen our understanding of the host–microorganism relationship in IR, we used multimodal techniques to conduct a comprehensive and extensive study investigating the interactions between the gut microbiome and metabolic diseases in humans. Although carbohydrate metabolism by the gut microorganisms has been suggested to influence the pathogenesis of obesity^3,4,25^ and prediabetes^6,8^, the actual mechanistic linkage has been elusive in humans owing to the lack of detailed metabolomic information. In this regard, the major strength of our approach is that we combine faecal metabolomics cataloguing more than 2,800 annotated metabolites with both microbiome and host pathology. This metabolome-based approach enabled us to identify the faecal metabolites related to IR, identify an association between faecal carbohydrates and low-grade inflammation of IR, and efficiently select candidate strains for functional validations in experimental settings (Extended Data Fig. 10e). Together, our study highlights the advantage of comprehensive omics strategy in exploring the involvement of microbial metabolism and their products in the pathogenesis of IR. Excessive monosaccharides have the potential to promote ectopic lipid accumulation while also activating immune cells, leading to low-grade inflammation and IR^40–42^. Fructose is a widely recognized risk factor for inflammation and IR due to its role in lipid accumulation^39^, whereas galactose has been shown to participate in the energy metabolism of activated immune cells^43^. Our in vivo studies confirm that *A. indistinctus* administration improves lipid accumulation and thereby IR, while simultaneously reducing intestinal monosaccharide levels (Fig. 4d). Nevertheless, we are aware that further mechanistic studies are needed to examine the kinetics of absorption and their effects on host metabolism. In particular, how *Alistipes* strains suppress carbohydrate metabolism is an intriguing question (for example, whether these bacteria per se inhibit carbohydrate metabolism, or whether they interact with other commensals), as it would directly open the possibility of a new therapeutic strategy. Given that *A. indistinctus* improved whole-body IS (Extended Data Fig. 9i), it would be important to investigate the involvement of insulin signalling not only in the liver but also in peripheral tissues, including skeletal muscle and adipose tissue, along with the accumulation of specific lipid molecules (such as ceramides and diacylglycerols) in these tissues. Such investigations hold the potential to shed light on the underlying mechanisms that contribute to *A. indistinctus*-mediated improvement of IR. Finally, two participants in the human study were unable to collect their faeces in the morning, which could potentially influence the outcomes due to the lack of stringent control over time-of-day and fasting conditions. We therefore believe that longitudinal studies incorporating a timely documentation of dietary habits are warranted to dissect the intricate impacts of microbial metabolism on the trajectory of diabetes and its complications while accounting for potential confounding factors.

## Methods

### Study participants and data collection

The study participants were recruited from 2014 to 2016 during their annual health check-ups at the University of Tokyo Hospital. The individuals included both male and female Japanese individuals aged from 20 to 75 years. The exclusion criteria were as follows: established diagnosis of diabetes, routine use of medications for diabetes and/or intestinal diseases, use of antibiotics within 2 weeks before sample collection and loss of 3 kg of body weight in the 3 months before sample collection. Written consent was obtained from the participants after a thorough explanation of the nature of the study at their health-checkups.

To normalize the participants’ clinical characteristics, we planned to recruit around 100 healthy individuals, 100 individuals with obesity (BMI ≥25, based on the Japanese definition) and 100 individuals with a prediabetic condition (FBG ≥110 mg dl^−1^ and/or HbA1c ≥6.0%) on the basis of their clinical data, and stopped recruiting when the number of participants almost reached the goal. The sample size was determined on the basis of previous metagenomics studies showing microbial signatures of diabetic patients^5,6^. We enrolled 112, 100 and 101 individuals for the normal, obese and prediabetic groups, respectively. The participants were provided with instructions to fast overnight before their visits, and all clinical information and blood samples were collected in the morning during their hospital visit. Blood samples were immediately centrifuged to collect plasma and then stored at −80 °C until the sample preparation and analysis. The participants were also instructed to collect faecal samples in the morning and were provided with guidance on how to collect and preserve faecal samples, along with a kit comprising a sampling tube and an ice pack. The faecal samples were then transported to the hospital either by refrigerated shipping or by the participants themselves. In both scenarios, the samples were delivered in a chilled state within 24 h after collection and stored at −80 °C until sample preparation and analysis. Consequently, 256 participants collected their faeces in the morning on the day of their hospital visit. As for the remaining participants, they collected their faeces in the morning between 2 days before and 7 days after their hospital visit, with the exception of 5 individuals who collected their faeces in the morning more than 7 days after their hospital visit, 2 individuals who reported collecting their faeces in the evening 1 day before their hospital visit, and 5 individuals who did not provide faecal samples. Moreover, two individuals withdrew from the study after enrolment. Thus, 306 individuals who underwent physical examination, laboratory tests, faecal sampling for faecal 16S rRNA pyrosequencing and metabolomic analyses, and plasma sampling for plasma metabolomic analyses were included for the analysis. Owing to the limited samples, faecal metagenomics data were available for 290 individuals; CAGE analysis data for 298 individuals; and plasma cytokine and insulin data for 282 individuals. The number of samples included in each analysis is described in the figure legends. The clinical study was approved by the institutional review board of RIKEN and The University of Tokyo and performed in accordance with the institutes’ guidelines.

Although we determined the criteria for enrolment, these criteria were not necessarily appropriate for subsequent analyses. For example, those in the prediabetes group were significantly leaner than those in the obese group (27.3 kg m^−2^ versus 25.2 kg m^−2^, *P* < 0.0001). Moreover, owing to the nature of the study participants (that is, those participated in regular health checkups), the blood glucose and HbA1c of the prediabetes group were significantly but only marginally higher than those of the obese group (FBG, 106 mg dl^−1^ versus 94 mg dl^−1^, *P* < 0.0001; and HbA1c, 6.2% versus 5.6%, *P* < 0.0001). We therefore reasoned that, in these subclinical conditions of diabetes, many metabolic traits may be overlapping between prediabetes and obesity groups and they do not necessarily capture their distinct features in metabolic and clinical continuums. This hinders us from distinguishing microbial and metabolomic characteristics directly related to human metabolic dysfunctions. We therefore considered that individual indices representing participants’ clinical conditions (that is, IR and MetS, as described below) may offer a better interpretation of the participants’ metabolic traits and data. Nevertheless, we observed consistent results even with the clinical criteria of obesity and prediabetes (Extended Data Fig. 2d).

### Phenotypic outcomes

IR is defined as HOMA-IR ≥2.5, as has been set for the Japanese population^13^. Similarly, normal IS was defined as HOMA-IR ≤1.6. HOMA-IR is calculated using the following formula: fasting plasma insulin (μU ml^−1^) × fasting plasma glucose (mg dl^−1^)/405. HOMA-IR values could be calculated for 282 individuals only, owing to the limited data of plasma insulin in some participants. MetS is diagnosed according to the Japanese criteria^44^, which require an abdominal circumference of ≥85 cm for male and ≥90 cm for female individuals and at least two out of the following three clinical abnormalities: (1) dyslipidaemia, defined as triglyceride levels of ≥150 mg dl^−1^ and/or HDL-C levels of <40 mg dl^−1^; (2) elevated blood pressure, defined as systolic blood pressure of ≥130 mmHg and/or diastolic blood pressure of ≥85 mmHg; and (3) impaired fasting glucose, defined as FBG levels of ≥110 mg dl^−1^. Individuals who meet the criteria of abdominal circumference but only one clinical abnormality were defined as pre-MetS, as reported previously^45^.

### Measurement of plasma cytokines

Plasma cytokines were measured using Human Adipokine Magnetic Bead Panel 2 (Millipore, HADK2MAG-61K) and Human Obesity Premixed Magnetic Luminex Performance Assay Kit (R&D, FCSTM08) according to the manufacturers’ instructions. Measurements below the lower detection limits were considered to be zero, and those above the upper detection limits were considered to be the highest values of analysed cytokines.

### Preparation for faecal samples

Aliquots (5 g) of faeces were blended with 30 ml methanol and filtrated with 100 µm of mesh filter to remove food residue after vigorous vortexing. The filtrate was centrifuged at 15,000*g* for 10 min at 4 °C and the supernatant (methanol extract) was used for metabolomics analysis. DNA of the faecal microbiome was extracted from the pellet.

### Extraction and measurement for hydrophilic metabolites of faecal and plasma samples

We followed the extraction process and gas chromatography-tandem MS (GC–MS/MS) measurement methods for water-soluble metabolites described previously^46^ with some modifications. In brief, a 10 μl aliquot of plasma was added to 150 μl methanol, 125 μl Milli-Q water, 15 μl internal standard solution (1 mM 2-isopropylmalic acid) and 60 μl CHCl_3_. For faecal samples, a 25 μl aliquot of methanol extract was added to 125 μl methanol, 150 μl Milli-Q water containing internal standard (100 μM 2-isopropylmalic acid) and 60 μl CHCl_3_. The solution was shaken at 1,200 rpm for 30 min at 37 °C. After centrifugation at 16,000*g* for 5 min at room temperature, 250 μl of the supernatant was transferred to a new tube and 200 μl of Milli-Q water was added. After mixing, the solution was centrifuged at 16,000*g* for 5 min at room temperature, and 250 μl of the supernatant was transferred to a new tube. The samples were evaporated dry using a vacuum evaporator for 20 min at 40 °C and lyophilized using a freeze dryer. Dried extracts were derivatized with 40 μl of 20 mg ml^−1^ methoxyamine hydrochloride (Sigma-Aldrich) dissolved in pyridine and shaken at 1,200 rpm for 90 min at 30 °C. The solution was then mixed with 20 μl of *N*-methyl-*N*-trimethylsilyl-trifluoroacetamide (MSTFA, GL Science) and incubated for 30 min at 37 °C with shaking at 1,200 rpm. After derivatization, the samples were centrifuged at 16,000*g* for 5 min at room temperature, and the supernatant was transferred to a glass vial. The analysis was performed using a GC–MS/MS platform on the Shimadzu GCMS-TQ8030 triple quadrupole mass spectrometer (Shimadzu) with a capillary column (BPX5, SGE Analytical Science). The GC oven was programmed as follows: 60 °C held for 2 min, increased to 330 °C (15 °C min^−1^), and finally 330 °C held for 3.45 min. GC was operated in constant linear velocity mode set to 39 cm s^−1^. The detector and injector temperatures were 200 °C and 250 °C, respectively. Injection volume was set at 1 μl with a split ratio of 1:30.

We followed the SCFA extraction and GC–MS/MS measurement methods as previously described^47^ with some modifications. A 90 μl aliquot of plasma was added to 10 μl Milli-Q water containing internal standards (2 mM [1,2-13C2]acetate, 2 mM [2H7]butyrate and 2 mM crotonate). For faecal samples, a 25 μl aliquot of methanol extract was added to 10 μl Milli-Q water containing internal standards and then centrifugally concentrated at 40 °C and reconstituted with 100 μl of Milli-Q water. Then, 50 μl of hydrochloric acid (HCl) and 200 μl of diethyl ether were added to the solution and mixed well. After centrifugation at 3,000*g* for 10 min, 80 μl of the organic layer was transferred to a glass vial and 16 μl *N*-tert-butyldimethylsilyl-*N*-trifluoroacetamide (MTBSTFA, Sigma-Aldrich) was added to derivatize the samples. The vials were incubated at 80 °C for 20 min and allowed to stand for 48 h before injection. The analysis was performed using a Shimadzu GCMS-TQ8030 triple quadrupole mass spectrometer with a capillary column (BPX5). The GC oven was programmed as follows: 60 °C held for 3 min, increased to 130 °C (8 °C min^−1^), increased to 330 °C (30 °C min^−1^) and finally 330 °C held for 3 min. The detector and injector temperatures were 230 °C and 250 °C, respectively. GC was operated in constant linear velocity mode set to 40 cm s^−1^. Injection volume was set at 1 μl with a split ratio of 1:30. The data were processed and concentration was calculated by LabSolutions Insight (Shimadzu).

Overall, 195 and 100 metabolites in the faecal and plasma samples, respectively, were detected by our GC–MS/MS platform and passed quality control. The values below the limit of detection were replaced with zero. Consequently, 110 faecal and 88 plasma metabolites that were detected (that is, above zero) in more than 75% of participants were included in subsequent analyses, for which they were combined into a common analysis pipeline and defined as hydrophilic metabolites.

### Lipidomics of faecal and plasma samples

The lipidomics analysis was performed according to a previously reported study^20^. Methanol, isopropanol, chloroform and acetonitrile of liquid chromatography (LC)–MS grade were purchased from Wako. Ammonium acetate and EDTA were purchased from Wako and Dojindo, respectively. Milli-Q water was purchased from Millipore (Merck). EquiSPLASH was purchased from Avanti Polar Lipids. Palmitic acid-d_3_ and stearic acid-d_3_ were purchased from Olbracht Serdary Research Laboratories.

For plasma lipid extraction, an aliquot of 20 μl of human plasma sample was added to 200 μl of methanol containing 5 μl of EquiSPLASH, 10 μM palmitic acid-d_3_ and 10 μM stearic acid-d_3_, and vortexed for 10 s. Then, 100 μl of chloroform was added and vortexed for 10 s. After incubation for 2 h at room temperature, the solvent tube was centrifuged at 2,000*g* for 10 min at 20 °C. A total of 200 μl of supernatant was transferred to an LC–MS vial (Agilent Technologies). For faecal lipid extraction, 50 μl of the methanol extract was added to 145 μl of methanol containing 5 μl of EquiSPLASH, 10 μM palmitic acid-d_3_ and 10 μM stearic acid-d_3_ in a 2 ml glass tube, and vortexed for 10 s. Then, 100 μl of chloroform was added and vortexed for 10 s. After incubation for 1 h at room temperature, 20 μl of water was added and vortexed for 10 s. After 10 min incubation at room temperature, the solvent was centrifuged at 2,000*g* for 10 min at 4 °C, and the supernatant was transferred to the LC–MS vial. All of the samples were divided into four batches for plasma analyses and five batches for faecal analyses, with 70–80 and 55–60 samples per batch after randomization, respectively. For each batch, a series of samples was prepared, and subsequent LC–MS/MS measurements were performed. A quality control sample was prepared by mixing the same volume of plasma from the first batch subjects. A procedure blank was prepared by using the same volume of water instead of a biological sample. The blank sample was analysed at the beginning and the end of each analysis batch, and the quality-control sample was injected every ten study samples.

The LC system consisted of a Waters Acquity UPLC system. Lipids were separated on an Acquity UPLC Peptide BEH C18 column (50 × 2.1 mm; 1.7 µm) (Waters). The column was maintained at 45 °C at a flow rate of 0.3 ml min^−1^. The mobile phases consisted of (A) 1:1:3 (v/v/v) acetonitrile:methanol:water with ammonium acetate (5 mM) and 10 nM EDTA; and (B) 100% isopropanol with ammonium acetate (5 mM) and 10 nM EDTA. A sample volume of 0.5−3 µl, depending biological samples, was used for the injection. The separation was conducted under the following gradient: 0 min, 0% B; 1 min, 0% B; 5 min, 40% B; 7.5 min, 64% B; 12 min, 64% B; 12.5 min, 82.5% B; 19 min, 85% B; 20 min, 95% B; 20.1 min, 0% B; and 25 min, 0% B. The sample temperature was maintained at 4 °C.

MS detection of lipids was performed on a quadrupole/time-of-flight mass spectrometer TripleTOF 6600 (SCIEX). All analyses were performed in high-resolution mode in MS1 (~35,000 full width at half-maximum) and the high sensitivity mode (~20,000 full width at half-maximum) in MS2. Data-dependent MS/MS acquisition (DDA) was used. The parameters were MS1 and MS2 mass ranges, *m*/*z* 70–1,250; MS1 accumulation time, 250 ms; MS2 accumulation time, 100 ms; collision energy, +40/−42 eV; collision energy spread, 15  eV; cycle time, 1,300 ms; curtain gas, 30; ion source gas 1, 40(+)/50(−); ion source gas 2, 80(+)/50(−); temperature, 250 °C(+)/300 °C(−); ion spray voltage floating, +5.5/−4.5 kV; declustering potential, 80 V. The other DDA parameters were dependent product ion scan number, 16; intensity threshold, 100 cps; exclusion time of precursor ion, 0 s; mass tolerance, 20 ppm; ignore peaks, within *m/z* 200; and dynamic background subtraction, true. The mass calibration was automatically performed using an APCI positive/negative calibration solution through a calibration delivery system.

MS-DIAL (v.4.48)^20,48^ was used with the following parameters: (data collection) retention time begin, 1.0 min; retention time end, 18 min; MS1 and MS2 mass range begin, 0 Da; MS1 and MS2 mass range end, 2,000 Da; MS1 tolerance, 0.01 Da; MS2 tolerance, 0.025 Da; (peak detection) minimum peak height, 3,000 amplitude; mass slice width, 0.1 Da; smoothing method, linear weighted moving average; smoothing level, 3 scan; minimum peak width, 5 scan; exclusion mass list, none; (identification) retention time tolerance, 1.5 min; MS1 accurate mass tolerance, 0.01 Da; MS2 accurate mass tolerance, 0.05 Da; identification score cut off, 70%; all lipid subclasses were used as the search space; (alignment) retention time tolerance 0.15 min; MS1 tolerance, 0.015 Da. The default values were used for other parameters. In faecal lipidomics, a total of 48,790 and 20,367 chromatographic peaks were detected in positive- and negative-ion mode data, respectively. Of these, 2,654 unique lipid molecules were annotated and semi-quantified in the MS-DIAL software program and used for further statistical analyses. Likewise, in plasma lipidomics, 1,469 and 2,167 chromatographic peaks were detected in positive- and negative-ion mode data, respectively, and 635 unique lipid molecules were annotated and semi-quantified. The semi-quantitative value of lipids was calculated by the internal standards according to the previous study^20^. The abbreviations of lipids are listed in Supplementary Table 27. Details of lipid subclass characterization follow the previous study^20^.

### Co-abundance clustering of metabolites

To generate co-abundance clusters, 110 hydrophilic metabolites and 2,654 lipid metabolites detected in more than 75% of participants were included. These metabolites were clustered based on their co-abundance using the R package WGCNA^49^ (v.1.72-1). The following parameters were used for the analysis. For hydrophilic metabolites, soft thresholding *β* = 12, minimum cluster size = 3, deep split = 4, cut height = 0.9999, PAM clustering = F. For lipid metabolites, soft thresholding *β* = 14, minimum cluster size = 20, deep split = 4, cut height = 0.999, PAM clustering = F. As soft thresholding of WGCNA was not able to cluster all of the metabolites, the remaining metabolites that did not fit the criteria were subsequently clustered on the basis of biweight midcorrelation. The following parameters were used for the secondary clustering. For hydrophilic metabolites, minimum cluster size = 3, deep split = 4, cut height = 0.9999, PAM clustering = F. For lipid metabolites, minimum cluster size = 6, deep split = 4, cut height = 0.999, PAM clustering = F. The clusters with biweight midcorrelation above 0.8 were merged. The first principal component (PC1) of each cluster was calculated using the moduleEigengenes command of WGCNA and used as the representative value of the cluster for further analyses. The representative classes of the clusters were described in Supplementary Tables 2 and 3. KEGG pathway enrichment analysis of CAGs was performed on MetaboAnalyst (v.5.0)^50^ using 84 metabolite sets based on the KEGG pathway. Hypergeometric test and false-discovery rate (FDR)-adjusted *P* values were used to test significance. The enrichment ratio was calculated as the ratio of actual metabolite number to the expected value in each pathway.

### Reanalysis of publicly available metabolomic data

To validate the associations between clinical markers and faecal metabolites, we used the metabolomic data of TwinsUK^17^ and HMP2 (ref. ^18^). The metabolome data of the TwinsUK cohort included 1,116 metabolites including 36 carbohydrates. The median (interquartile range) of age and BMI were 65 years (60–71 years) and 25.4 (22.8–28.8), and the proportion of males was 6.6%. As reported previously^17^, the metabolite levels were scaled by run-day medians. The data were then log-transformed and scaled. For regression analyses, we filtered out the metabolites detected in less than 50% of participants; as a result, 759 metabolites including 29 carbohydrates were used for further analyses. The record of BMI and HOMA-IR were used for phenotypic outcomes. For BMI, we retrieved 786 samples measured on the same day of faecal collection. For HOMA-IR, plasma glucose and insulin obtained in the same year of the faecal collection were used for the following calculation: plasma glucose (mM) × insulin (pM)/6.945/22.5. We identified 550 individuals who underwent both faecal collection and glucose and insulin measurement in the same year and included them in the analysis. The HMP2 data were obtained from the Inflammatory Bowel Disease Multi’omics Database (https://ibdmdb.org/). Among the 26 out of 106 samples from non-IBD control, BMI data were available for 20 samples. We further excluded four individuals aged <10 years. As HMP2 is a longitudinal study, only the first faecal sampling for metabolomics was used for the current analysis to avoid redundancy. The intensity of fructose, glucose and/or galactose was log-transformed and scaled.

### DNA extraction from faecal samples

DNA extraction was performed according to a protocol described previously^47^ with slight modifications. Before DNA extraction, the faecal pellet was washed once with PBS and suspended in a 10 mM Tris-HCl/20 mM EDTA buffer (pH 8.0). Lysozyme (Sigma-Aldrich), achromopeptidase (Wako) and proteinase K (Merck) were subsequently added to the samples for cell lysis. DNA was recovered by a phenol–chloroform extraction method. To purify the extracted DNA, RNA was digested with RNase (Nippon Gene). DNA was then precipitated in a solution containing polyethylene glycol 6000 (Hampton Research). The DNA concentration was quantified using Quant-iT PicoGreen (Thermo Fisher Scientific).

### 16S rRNA gene sequencing and taxonomic assignment

The hypervariable V1–V2 region of the 16S rRNA gene was amplified by PCR using barcoded primers. PCR amplicons were purified using AMPure XP magnetic purification beads (Beckman Coulter), and quantified using the Quant-iT PicoGreen dsDNA Assay Kit (Life Technologies Japan). Equal amounts of each PCR amplicon were mixed and then sequenced using the MiSeq (Illumina) system.

On the basis of sample-specific barcodes, reads were assigned to each sample using bcl2fastq. Next, the reads lacking both forward and reverse primer sequences were removed using BLAST and parasail followed by trimming of both primer sequences. Data were further denoised by removing reads with average quality values of <25 and possible chimeric sequences. Reads with BLAST match lengths of <90% with the representative sequence in the 16S databases (described below) were considered to be chimeras and were removed. The filter-passed reads were used for further analysis. The 16S database was constructed from three publicly available databases: the Ribosomal Database Project (RDP; v.10.27), CORE (http://microbiome.osu.edu/) and a reference genome sequence database obtained from the NCBI FTP site (ftp://ftp.ncbi.nih.gov/genbank/, December 2011).

Operational taxonomic unit (OTU) clustering and UniFrac analysis from the filter-passed reads, 3,000 high-quality reads per sample were randomly chosen. All reads (the number of samples × 3,000) were then sorted according to their average quality value and grouped into OTUs using UCLUST (http://www.drive5.com/) with a sequence-identity threshold of 97%. The representative sequences of the generated OTUs were processed for homology search against the databases mentioned above using the GLSEARCH program for taxonomic assignments. For assignment at the phylum, genus and species levels, sequence similarity thresholds of 70%, 94% and 97% were applied, respectively.

### Shotgun metagenomic sequencing

Metagenome shotgun libraries (insert size of 500 bp) were prepared using the TruSeq Nano DNA kit (Illumina) and sequenced on the Illumina NovaSeq platform. After quality filtering, reads mapped to the human genome (HG19) or the phiX bacteriophage genome were removed. For each individual, the filter-passed NovaSeq reads were assembled using MEGAHIT (v.1.2.4). Prodigal (v.2.6.3) was used to predict protein-coding genes (≥100 bp) in the contigs (≥500 bp) and singletons (≥300 bp). Finally, 6,458,217 non-redundant genes were identified in the 290 samples by clustering the predicted genes using CD-HIT with a 95% nucleotide identity and 90% length coverage cut-off. Functional assignment of the non-redundant genes was performed using DIAMOND (*e*-value ≤ 1.0 × 10^−5^) against the Kyoto Encyclopedia of Genes and Genomes (KEGG) database (release 2019-10-07) to obtain the KEGG orthologues. The genes with the best hit correlating to eukaryotic genes were excluded from further analysis.

### Quantification of annotated genes in human gut microbiomes

For taxonomic assignment of metagenomic reads, 1 million filter-passed reads were processed for mOTU analysis (v.2.5.1)^51^ to obtain the relative abundance at the species level. To functionally annotate the predicted genes, 1 million filter-passed metagenomic reads per individual were mapped to the combined reference gene set consisting of non-redundant genes identified in this study, JPGM^52^ and IGC^53^ using Bowtie2 with a 95% identity cut-off. Multi-mapped reads, that is, the reads that mapped to multiple genes with identical scores, were normalized to the proportion of the number of other reads that uniquely mapped to these genes, according to a strategy outlined in a previous report^52^. The proportion of KEGG orthologues was calculated from the number of reads mapped to them. For the enrichment analysis of KEGG pathways, the significantly and positively (negatively) associated KEGG orthologue gave +1 (−1) for all of the upstream pathways linked to the KEGG orthologue, and the points were summarized as the ratio to the number of KEGG orthologues in the pathway. For the KEGG-orthologue-level analyses of PTS, those including ‘phosphotransferase system (PTS)’ in the KEGG pathway (02060) were selected for the following correlation analyses. In the analyses of glucosidases, ‘glycoside hydrolases’ defined in the CAZy database on the basis of EC numbers^54^ were selected. We further selected those included in ‘starch and sucrose metabolism’ in the KEGG pathway (00500). We defined intracellular glucosidase by their substrate described in the KEGG pathway map; those cleave phosphorylated carbohydrates were recognized as intracellular, and the rest of the genes were recognized to possess extracellular enzymatic activities. The pathways were further summarized into carbohydrate metabolism (09101), amino acid metabolism (09105), lipid metabolism (09103) and membrane transport (09131) on the basis of the KEGG Orthology database.

### Comparison of KEGG organism genomes

The list of KEGG organisms used for this genome analysis is listed in Supplementary Table 28. All KEGG organisms from genera *Alistipes*, *Bacteroides*, *Flavonifractor*, *Blautia*, *Dorea* and *Coprococcus*, which showed the top three positive or negative correlations with faecal carbohydrates in Fig. 2d, were selected for this analysis. The lists of genes involving the ‘starch and sucrose metabolism’ pathway (00500) in these KEGG organisms were extracted using the R package KEGGREST (v.1.32.0). The representative protein sequences of *Blautia hydrogenotrophica* strain 2789STDY5608857 (taxonomy ID 53443), *Dorea longicatena* strain 2789STDY5608851 (taxonomy ID 88431) and *Dorea formicigenerans* strain ATCC 27755 (taxonomy ID 411461) were downloaded from the NCBI Datasets (https://www.ncbi.nlm.nih.gov/datasets/genomes/). KEGG annotation of these protein sequence files was performed using BlastKOALA (https://www.kegg.jp/blastkoala/) with ‘Bacteria’ used as the taxonomy group. The presence of KEGG orthologues relating to extracellular glycoside hydrolases in starch and sucrose metabolism pathways shown in grey in Extended Data Fig. 6f was summarized.

### RNA extraction from PBMC

Blood samples were collected in Vacutainer CPT tubes (Becton Dickinson) and mixed with the anticoagulant by gently inverting the tubes 8 to 10 times. After centrifugation of the blood for 30 min at 1,500*g*, PBMCs were isolated as a diffuse layer above the gel. The plasma was removed, and the PBMCs were collected in conical tubes with 500 μl RNAlater (Thermo Fisher Scientific). The conical tubes were centrifuged at 1,000*g* at room temperature for 3 min to pellet the cells and the supernatant was discarded. The RNA was then isolated using the Maxwell 16 LEV simplyRNA Blood Kit (Promega) according to the manufacturer’s instructions. The quality of the RNA was assessed using Bioanalyzer (Agilent), as recommended by the manufacturer. The RNAs were quantified using the GloMax plate reader (Promega) and Quant-iT RiboGreen RNA Assay Kit (Thermo Fisher Scientific).

### CAGE analysis

The CAGE libraries were constructed according to the dual-index nanoCAGE protocol, a template-switching-based variation of the standard CAGE protocol designed for low quantities of RNA^55,56^. cDNA libraries were prepared with RNA extracted from PBMC samples and sequenced on the Illumina HiSeq 2000 (50 bp paired-end). The sequenced reads were processed with the MOIRAI pipeline^57^: low quality and rDNA reads were first removed, then the remaining reads were mapped to the human genome version hg38 patch 1 using BWA v.0.5.9 (r16). The mapped reads were overlapped with the FANTOM5 robust promoter set (http://fantom.gsc.riken.jp/5/datafiles/latest/extra/CAGE_peaks/) and mapped to the nearest GENCODE v.27 annotations within (500 bp)^58,59^. The mapped reads falling under each FANTOM5 CAGE cluster were summed to produce the raw expression counts. Expression counts were converted to counts per million (CPM), and CAGE clusters expressed in less than 100 samples with at least 1 CPM and greater than 1 sample with at least 10 CPM were removed from further analysis. For each sample, the richness index was calculated using the R package vegan’s rarefy function with a subsample size of 100 on the filtered raw counts. Samples with a read library size of less than 1,000,000 and a number of unique CAGE clusters of <11,000 and richness less than 44 were removed as outliers, with the thresholds selected from visual inspection of the respective distributions. Cell type specificities of promoters of interest were determined using the FANTOM5 hg38 human promoterome view.11 in the ZENBU genome browser (https://fantom.gsc.riken.jp/zenbu/). Top-hit cells for analysed promoters were described. For cell-type gene set enrichment analysis of genes significantly associated with IR, annotated genes were analysed using Enrichr^60,61^ and the Human Gene Atlas database^60^, and the results of cell types with *P*_adj_ < 0.05 were selected. The Enrichr combined score is defined as: *c* = log[*p*] *z*, where c is the combined score, *p* is the *P* value based on Fisher’s exact test and *z* is the *z*-score^60^.

### Metabolite measurement in bacterial culture

The following strains were used for this culture analysis: *A. indistinctus* (JCM16068), *A. finegoldii* (JCM16770), *Alistipes putredinis* (JCM 16772), *B. thetaiotaomicron* (JCM 5827), *Bacteroides xylanisolvens* (JCM 15633), *Bacteroides ovatus* (JCM 5824), *Bacteroides caccae* (JCM 9498), *Parabacteroides merdae* (JCM 9497), *Parabacteroides distasonis* (JCM 5825), *D. formicigenerans* (JCM 31256), *D. longicatena* (JCM 11232), *B. hydrogenotrophica* (JCM 14656), *Blautia producta* (BP, JCM 1471), *Coprococcus comes* (JCM 31264), *Faecalibacterium prausnitzii* (JCM 31915), *Flavonifractor plautii* (JCM 32125), *Clostridium spiroforme* (JCM1432), *Coriobacterium glomerans* (JCM 10262), *Roseburia hominis* (JCM 17582), *Adlercreutzia equolifaciens* subsp. *equolifaciens* (JCM 14793), *Eggerthella lenta* (JCM 9979) and *Collinsella aerofaciens* (JCM 10188). All strains were obtained from RIKEN BioResource Research Center. All of the strains were cultivated in EG medium (JCM Medium No. 14) supplemented with 5% Fildes extract prepared by pepsin-digested horse blood instead of horse blood itself. For measurement of metabolites in bacterial culture, 60 μl of the bacterial culture grown in the EG medium was inoculated into 3 ml of the experiment medium (EG medium) and cultivated for 24 h. The samples were centrifuged, and the cell-free supernatant was collected for analysis. GC–MS was performed to measure hydrophilic metabolites as described above. We identified 261 metabolites by the analysis and used 198 metabolites observed in at least 30% of samples for the following analyses.

### Animal experiments

C57BL6/N male mice aged 6 weeks were purchased from CLEA Japan. They were randomly assigned to either the control or treatment group and housed in a conventional animal facility of Yokohama City University. The mice were fed Quick Fat (CLEA Japan) for 3 weeks before bacterial administration and continued to be fed for 3 weeks during bacterial challenges. *A. indistinctus* (JCM16068), *A. finegoldii* (JCM16770), *B. thetaiotaomicron* (JCM 5827), *B. xylanisolvens* (JCM 15633), *P. merdae* (JCM 9497), *F. prausnitzii* (JCM 31915) and *C. spiroforme* (JCM1432) were used to broadly compare the efficacy of bacterial administration on the animal model. These strains were cultivated in EG medium overnight, and the concentration was adjusted to 2.5 × 10^8^ CFU per ml by PBS. The bacteria and PBS, a negative control, were orally administered to the mice at a dose of 200 μl per mouse. The bacteria and PBS as the vehicle control were provided 3 times a week for 3 or 4 weeks. Body mass was measured before oral gavage. Postprandial blood glucose measurement and insulin tolerance test were performed 3 weeks after the initiation of bacterial challenges. After the insulin tolerance test, the mice were subjected to 5 h fasting before insulin injection, and 0.85 U kg^−1^ human regular insulin (Eli Lilly) was subsequently administered intraperitoneally. The intraperitoneal glucose tolerance test was performed 4 weeks after the initiation of bacterial challenges. The mice were subjected to 5 h fasting before glucose infusion, and 2.0 g per kg glucose (Nacalai Tesque) was administered intraperitoneally. In both experiments, the blood glucose was collected from the tail vein and serially measured using GLUCOCARD G Black (Arkray). For the necropsy, the mice were euthanized by isoflurane (MSD), and the fat mass of perigonadal and mesenteric fats was measured. Blood was drawn through cardiac puncture after the anaesthesia. HDL-C (Wako), triglycerides (Wako) and adiponectin (Otsuka) were measured in accordance with the manufacturers’ instructions. The Yokohama City University animal facility is maintained under a 12 h–12 h light–dark cycle at 24 ± 1.5 °C and 55 ± 10% humidity.

To assess the metabolism, dietary intake and locomotor activity of mice, C57BL6/N male mice at the age of 6 weeks were purchased from CLEA Japan and were maintained in a vinyl isolator of SPF animal facility at RIKEN Yokohama branch. Using the same experimental protocol in the conventional condition, the mice were fed Quick Fat (CLEA Japan) for 3 weeks before bacterial administration and continued to be fed the diet during bacterial challenges and metabolic measurement. We gave three oral gavages of *A. indistinctus* or PBS (vehicle control) every other day and then placed the mice individually in acrylic cages. We further gave one oral gavage 2 days after the start of individual housing. Their metabolic activity, dietary intake and physical activity were subsequently monitored. There was no significant difference in body mass at the start of metabolic measurement (mean ± s.d. of body mass were 25.7 ± 2.6 g and 26.1 ± 1.4 g in the vehicle and *A. indistinctus* groups, respectively). Oxygen and carbon dioxide concentration was measured using the ARCO-2000 system, an open-circuit metabolic gas analysis system with a mass spectrometer (Arco Systems). VO_2_, VCO_2_, energy expenditure, fat oxidation rate, carbohydrate oxidation rate and respiratory quotient were calculated within the system. Dietary intake and physical activity were simultaneously monitored through ACTIMO-100M and MFD-100M (Shinfactory). The differences in diurnal variation were tested using two-way mixed ANOVA, and *P* values for interactions between time and group were reported. The RIKEN animal facility is maintained under a 12 h–12 h light–dark cycle at 23 ± 2 °C and 50 ± 10% humidity. The sample size was determined on the basis of our preliminary experiments. Bacterial administration and body mass measurements were performed by an independent researcher who was not involved in the grouping and outcome assessments. All experimental procedures were approved by the Institutional Animal Care and Use Committee of the RIKEN and Yokohama City University and performed in accordance with the institutes’ guidelines.

### Western blot analysis of phosphorylated AKT

To analyse phosphorylation of AKT (p-AKT) at Ser473, the mice administered with *A. indistinctus*, *A. finegoldii* and PBS (vehicle control) 3 times a week for 4 weeks were subjected to 6 h fasting before the insulin injection, and 0.85 U kg^−1^ human regular insulin (Eli Lilly) was subsequently administered from the inferior vena cava. The liver, epididymal fat (eWAT) and gastrocnemius muscle were subsequently collected 5 min after the insulin injection, weighed and snap-frozen by liquid nitrogen. To prepare the lysates for western blotting, the tissues were homogenized in buffer A (25 mM Tris-HCl, pH 7.4, 10 mM sodium orthovanadate, 10 mM sodium pyrophosphate, 100 mM sodium fluoride, 10 mM EDTA, 10 mM EGTA and 1 mM phenylmethylsulfonyl fluoride). Thereafter, the lysates were resolved on 10% SDS–PAGE. Phosphorylated or total protein of AKT was isolated by immunoblotting using specific antibodies after the tissue lysates were resolved by SDS–PAGE and transferred to a Hybond-P PVDF transfer membrane (Amersham Biosciences). Bound antibodies were detected with HRP-conjugated secondary antibodies using ECL detection reagents (Amersham Biosciences). Rabbit polyclonal antibodies directed against AKT and p-AKT (Ser473) were purchased from Cell Signaling Technology. Precision Plus Protein All Blue Standards (Bio-Rad) were used for the molecular mass markers.

### Hyperinsulinaemic–euglycaemic clamp test

The protocol has been published elsewhere^62,63^. Mice administered with *A. indistinctus* or PBS (vehicle control) for 5 to 6 weeks were used for the experiment. Jugular vein catheterization was performed 1 day before the clamp test. In brief, a mouse was anaesthetized with isoflurane (MSD), and the right jugular vein was exposed. A double-channel catheter was subsequently inserted to the vein. The next day, the mice were subjected to 4 h fasting before the clamp test. Human regular insulin (Eli Lilly) was intravenously administered at 7.5 mU kg^−1^ min^−1^, and the blood glucose levels were monitored every 5  min for 120 min. 50% glucose solution containing 6,6-d_2_-glucose (Sigma-Aldrich) was simultaneously infused to keep blood glucose levels around 100 to 120 mg dl^−1^. To separate the plasma, approximately 25 μl of blood was also drawn from tail vein at 0, 90, 105 and 120 min, placed into a tube containing 2 μl of heparin (Mochida Pharmaceutical) and centrifuged at 12,000*g* at 4 °C for 5 min. The plasma levels of glucose and 6,6-d_2-_glucose were measured using GC–MS. In brief, a 5 µl aliquot of plasma was extracted and derivatized with methoxyamine hydrochloride (Sigma-Aldrich) and *N*-methyl-*N*-(trimethylsilyl)trifluoroacetamide (GL Sciences), as previously described^46^. The analysis was performed using a GC–MS/MS platform on a Shimadzu GCMS-TQ8040 triple quadrupole mass spectrometer (Shimadzu) with a capillary column (BPX5) (SGE Analytical Science/Trajan Scientific and Medical). The programme of GC–MS/MS analysis was previously described^46^ with minor modifications. We integrated each derivative peak to obtain mass isotopomers of glucose for the following ions: *m*/*z* 319.1, 320.1 and 321.1. The glucose infusion rate was determined as the infusion rate at 90, 105 and 120 min. The rate of glucose disappearance was determined on the basis of the plasma levels of 6,6-d_2_-glucose and total glucose using a non-steady-state equation as described previously^63,64^ and considered as the whole-body glucose disposal after insulin stimulation. Hepatic glucose production was determined as the subtraction of glucose disappearance rate and glucose infusion rate.

### Analysis of triglyceride contents in the liver

For the necropsy, the mice were anesthetized using isoflurane (MSD), and the left half of liver was dissected, weighed and frozen in liquid nitrogen. The extraction of triglyceride contents from the liver tissue has been reported elsewhere^62,64^. In brief, the samples were homogenized in buffer A (25 mM Tris-HCl at pH 7.4, 10 mM sodium orthovanadate, 10 mM sodium pyrophosphate, 100 mM sodium fluoride, 10 mM EDTA, 10 mM EGTA and 1 mM phenylmethylsulfonyl fluoride) and mixed with chloroform/methanol (2:1, v/v). The mixture was shaken for 15 min, centrifuged and the organic layer was collected. The extraction step was repeated three times. The collected samples were evaporated and resuspended in 1% Triton X-100/ethanol. The triglyceride content was assessed using Triglyceride E-test Wako (Wako) according to the manufacturer’s instructions.

### Statistical methods and comparisons

For general statistical comparisons, two-sided Wilcoxon rank-sum tests were used for two-group comparisons, Kruskal–Wallis tests followed by Dunn’s post hoc analysis were used for comparisons of more than two groups, and Fisher’s exact tests were used for comparison of categorical variables. For general correlation analyses, Spearman’s rank correlation in the function corr.test of the R package psych v.2.1.6 was used. For partial correlation analyses, partial Spearman’s rank correlation in the function pcor.test of the R package ppcor v.1.1 was used. To predict the metabolite levels and their CAGs (Fig. 1b,d and Extended Data Figs. 2c,d and 3a), rank-based regression analyses were performed using the function rfit of the R package Rfit (v.0.24.2)^65^. For the ordinal independent variables (that is, IR, MetS, and original categories with obese and prediabetes), IS, no MetS, and healthy categories were considered as the references, respectively, and the coefficients and *P* values for other categories were calculated against these reference categories. For the analyses involving generalized linear models (GLM) such as Fig. 2b and Extended Data Figs. 5i and  6c, the dependent variables were assumed to follow a Gamma distribution and arcsine square root transformation was applied to the relative-abundance values of microbiota and KEGG orthologues. To enhance comparability, the standardized coefficient was also calculated by standard deviations of dependent and independent variables using the function lm.beta of the R package QuantPsyc v.1.5 in Extended Data Fig. 5i. In the reanalysis of TwinsUK data, we fitted generalized linear mixed-effects models with age, sex, zygosity and BMI as fixed effects and sample collection year as a random effect using the function glmer of R package lme4 v.1.1-27.1 to estimate the associations between HOMA-IR and faecal carbohydrate metabolites (Extended Data Fig. 3b,c). Similarly, in the reanalysis of HMP2 data, we fitted a generalized linear mixed-effects model with consent age and sex as fixed effects and sample collection site as a random effect to estimate the associations between BMI and faecal fructose, glucose and/or galactose (Extended Data Fig. 3d). To analyse the associations between the participants’ clusters and clinical markers in Fig. 2b, the clusters were reordered before regression analyses according to their proportion of individuals with IR, where cluster C showing the lowest proportion of IR was set as the reference. To calculate the KEGG pathway enrichment associated with the participant clusters (Fig. 2e and Extended Data Fig. 6a,b), the KEGG orthologues were compared between each cluster and the remaining three clusters using a two-sided Wilcoxon rank-sum test, and significant (*P*_adj_ < 0.05) KEGG orthologues were summarized into the pathway level (Supplementary Table 16). For comparison of metabolites in bacterial cultures (Extended Data Fig. 8), one-way ANOVA followed by Tukey’s post hoc test was performed, followed by multiple testing corrections based on the Benjamini–Hochberg procedure. For comparisons of time-series data such as insulin tolerance test, two-way repeated-measures ANOVA was used and the between-group difference was analysed by estimated marginal means. *P* *<* 0.05 was considered to be significant. To analyse the body mass change in animal experiments, ANCOVA analysis was performed to adjust baseline body mass (that is, body mass change as a dependent variable and group and baseline body mass as independent variables). We also validated the assumption of this ANCOVA model, that is, homogeneity of regression slopes, homogeneity of variances and normality of residuals. For multiple-testing corrections, *P* values were corrected using the Benjamini–Hochberg procedure using the R function p.adjust. *P*_adj_ < 0.05 was used as a cut-off unless otherwise specified. All data were collected using Microsoft Excel 2016. All statistical and graphical analyses were conducted using R v.4.1.1 using R studio v.1.4.1717, unless otherwise specified.

### ROC curve analysis of omics datasets

To analyse ROC curves of omics datasets, the datasets of faecal metabolomics, including hydrophilic and lipid metabolites, faecal 16S rRNA gene sequencing at the genus level, faecal metagenome consisting of KEGG orthologues and clinical metadata, were included. We first selected feature variables in each dataset, that is, the best explaining variables in the given dataset, using the minimum redundancy maximum relevance (mRMR) algorithm^15^. The function mRMR.classic of the R package mRMRe v.2.1.2.1 was used for the calculation. The datasets were square-root-transformed before mRMR calculation. We selected 5 to 50 variables in 5 increments as the maximum number of genera was 50. Using the selected variables, we next established random-forest models using the R package caret v.6.0-88 to classify the individuals into IR or not. Specifically, the results of mRMR were split into train and test datasets in a 3:1 ratio. The generated random-forest models were evaluated using a tenfold cross-validation method and applied to the test datasets to obtain probability scores. The accuracy of each classification model was described by the AUC of ROC curves using the R package pROC v.1.17.0.1.

### Construction of microorganism–metabolite networks

To construct the co-abundance networks of genus-level bacteria, we selected 28 genus-level microorganisms that were observed in more than 40% of the participants and calculated the correlations using the R package CCREPE (compositionality corrected by renormalization and permutation)^66^ v.1.28.0 with Spearman’s correlations and the default settings. Interactions with *P*_adj_ < 0.05 were selected for further analysis. Bacteria that exhibited a positive correlation with one another were determined to be members of an independent co-abundance microbial group, except for the interaction between *Bacteroides* and *Robinsoniella*. We decided to categorize *Robinsoniella* into the *Blautia* and *Dorea* group owing to its stronger correlation with *Blautia* in comparison to *Bacteroides*, both of which showed the highest centrality within their respective networks. Those weakly associated with each other or negatively associated with the members of other CAGs were classified as miscellaneous (Extended Data Fig. 5f). To characterize the microbial profiles of the study participants, the individuals were clustered on the basis of the abundance of 28 genera, which includes 20 genera in co-abundance microbial groups identified with CCREPE and 8 unclustered genera, using the ward.D function of the R package pheatmap v.1.0.12. Four distinct clusters of participants were determined, and the proportion of IR was compared using Fisher’s exact tests. Microorganism–metabolite networks were constructed on the basis of the correlations between the 28 genera observed in at least 40% of samples and the faecal metabolites, including all hydrophilic metabolites (*n* = 110) and bacteria-related lipid metabolites (*n* = 259). Bacteria-related metabolites were defined according to previous reports^20,21^. The following classes were selected: DGDG, PE-Cer, MGDG O, FAHFA, Cer-AS, Cer-BDS, NAGly, NAGlySer, PI-Cer, SL, AcylCer, bile acids, DGDG O and AAHFA. Positive and negative Spearman’s correlations with *P*_adj_ < 0.05 were separately depicted in the networks. The networks were visualized using Cytoscape (v.3.7.0)^67^.

### Construction of cross-omics networks

To construct and visualize a correlation-based network of omics data, we first analysed IR-associated host signatures using plasma cytokines, plasma metabolites and CAGE promoter expression data. We identified the significant host markers through the following models: (1) GLM with a gamma distribution: HOMA-IR as a dependent variable and host markers, age and sex as independent variables; (2) logistic regression model: IR (HOMA-IR ≥ 2.5 = 1, HOMA-IR ≤ 1.6 = 0) as a dependent variable and significant host markers in the model 1, age and sex as independent variables. In both models, host markers with *P*_adj_ < 0.05 were considered to be significant. We finally identified 6, 21 and 36 significant associations from plasma cytokines, plasma metabolites and CAGE promoter expression data, respectively (Supplementary Tables 19–21). In terms of bacteria, 20 genera with significant interactions between each other, which were identified with CCREPE as shown in Extended Data Fig. 5f, were included. In terms of faecal metabolites, 15 carbohydrates associated with IR in the CAG analysis as shown in Fig. 1b were included. Pairwise partial Spearman’s rank correlations adjusted by age, sex, BMI and FBG between all given factors were calculated with the R package ppcor v.1.1. The correlations with *P*_adj_ < 0.05 were selected for visualization. The size of nodes was determined as the ratio of median abundance in IR over IS. As the median values of genera *Robinsoniella* and *Rothia* were zero, these elements were removed from the visualization. The width of lines was determined as the absolute value of partial Spearman’s coefficient. The networks were visualized using Cytoscape v.3.7.0. as in the microorganism–metabolite networks described above.

### Explained variance of plasma cytokines by omics data

To assess the explained variance of ten plasma cytokines, we established random-forest models using the R package caret v.6.0-88 to predict the plasma cytokine levels using 15 IR-associated faecal carbohydrates identified in Fig. 1b; 20 genera with significant interactions with each other that were identified in Fig. 2a; 21 IR-associated plasma hydrophilic metabolites (Supplementary Table 20); or 36 IR-associated CAGE promoters (Supplementary Table 21). Plasma cytokines were log_10_-transformed and scaled before the regression analyses. The data were split into train and test datasets at a 4:1 ratio. The generated random-forest models were evaluated using a tenfold cross-validation method and applied to the test datasets to obtain predictions. The explained variance shown as *R*^2^ was calculated as its definition: 1 − sum(test − predict)^2^/sum(test − mean(test))^2^. The negative values were considered as zero.

### Causal mediation analysis

To infer the effects of plasma cytokines on in silico causal relationships between faecal carbohydrates and IR markers (HOMA-IR, BMI, triglycerides and HDL-C), we performed causal mediation analysis using the R package mediation (v.4.5.0)^38^. As previously reported^68^, we first screened significant associations (*P*_adj_ < 0.05) between 15 IR-associated faecal carbohydrates and four IR markers, and significant associations between ten plasma cytokines and four IR markers. Age and sex were included as independent variables in both models. We then performed causal mediation analyses with the following models: (1) Mediator models: cytokine ~ metabolite + age + sex; (2) outcome models: IR marker ~ metabolite + age + sex + cytokine. In both models, faecal carbohydrate and plasma cytokine values were scaled before the analyses, and GLM with Gaussian distribution was used. A nonparametric bootstrap procedure was used to calculate the significance, followed by multiple testing corrections using the R function p.adjust. Average causal mediation effects and average direct effects with *P*_adj_ values from representative models are reported in Extended Data Fig. 7d, whereas all of the results including the total effects and proportion mediated are reported in Supplementary Table 23.

### Reporting summary

Further information on research design is available in the Nature Portfolio Reporting Summary linked to this article.

## Online content

Any methods, additional references, Nature Portfolio reporting summaries, source data, extended data, supplementary information, acknowledgements, peer review information; details of author contributions and competing interests; and statements of data and code availability are available at 10.1038/s41586-023-06466-x.

## Supplementary information

Supplementary Fig. 1 Raw images of blotting membranes. a,b, The blotting membranes images of p-AKT and total AKT in the liver (a) and epidydimal fat (b). Molecular mass (kDa) is shown on the left. Relating to Extended Data Fig. 9k.

Reporting Summary

Supplementary Tables Supplementary Tables 1–28.

## Data Availability

Raw sequencing data of faecal microbiota have been deposited at the DNA Data Bank of Japan’s BioProject (https://www.ddbj.nig.ac.jp/bioproject/index-e.html) under accession number PRJDB11444. Raw metabolomic data have been deposited at the RIKEN DROP Met (http://prime.psc.riken.jp/menta.cgi/prime/drop_index) under index number DM0037. Raw CAGE sequencing data are deposited at the Japanese Genotype-phenotype Archive of National Bioscience Database Center (https://humandbs.biosciencedbc.jp/en/) under accession number JGAS000569. The following publicly available databases were used in this study: Ribosomal Database Project (https://www.canr.msu.edu/cme/resources#:~:text=RIBOSOMAL%20DATABASE%20PROJECT,J), CORE (http://microbiome.osu.edu/), a reference genome sequence database obtained from the NCBI FTP site (ftp://ftp.ncbi.nih.gov/genbank/, December 2011), UCLUST (http://www.drive5.com/), the KEGG Orthology database (https://www.genome.jp/kegg/ko.html), glycoside hydrolase family classification in the CAZy database (http://www.cazy.org/Glycoside-Hydrolases.html), the Inflammatory Bowel Disease Multi’omics Database (https://ibdmdb.org/) and the Human Gene Atlas Database associated with Enrichr (https://maayanlab.cloud/Enrichr/). Source data are provided with this paper.

## Source data

Source Data Fig. 1
Source Data Fig. 4
Source Data Extended Data Fig. 5
Source Data Extended Data Fig. 9
Source Data Extended Data Fig. 10
