# Supplementary material for: Gut microbial carbohydrate metabolism contributes to insulin resistance
Source: Nature. 2023 Aug 30;621(7978):389–95. doi: 10.1038/s41586-023-06466-x (PMC10499599; doi:10.1038/s41586-023-06466-x)
Supplement: Supplementary file 1 — Raw images of blotting membranes. a,b, The blotting membranes images of p-AKT and total AKT in the liver (a) and epidydimal fat (b). Molecular mass (kDa) is shown on the left. Relating to Extended Data Fig. 9k. [file 41586_2023_6466_MOESM1_ESM.pdf]

nature portfolio

https://doi.org/10.1038/s41586-023-06466-x

Supplementary information

Gut microbial carbohydrate metabolism contributes to insulin resistance

In the format provided by the authors and unedited

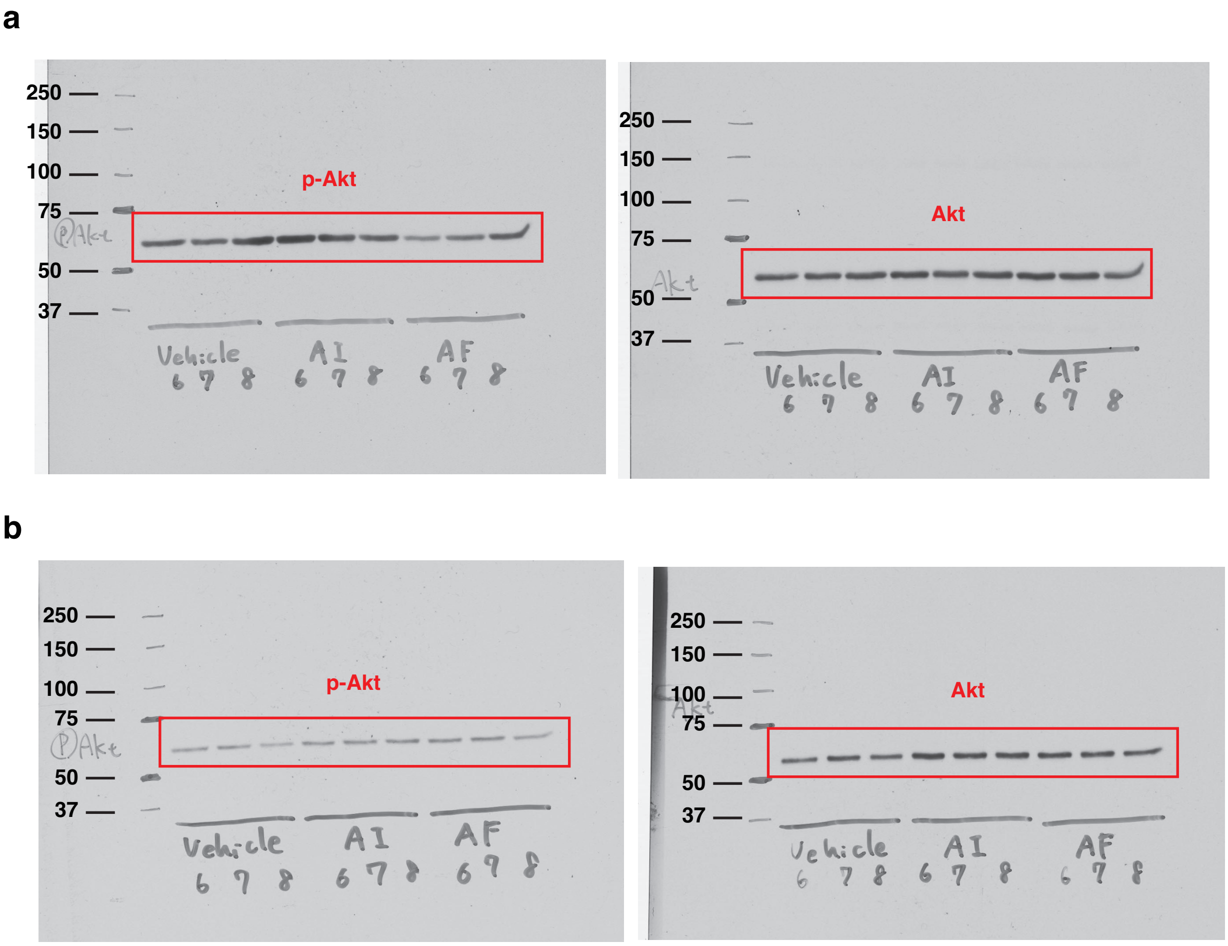

Supplementary Fig. 1. Raw images of blotting membranes. a, **b**, The blotting membranes images of phosphorylated (p-Akt) and total Akt in the liver (**a**) and epidydimal fat (**b**). Molecular weight (kDa) is shown on the left. Relating to Extended Data Fig. 9k.
