# Supplementary material for: Gut microbial carbohydrate metabolism contributes to insulin resistance
Source: Nature. 2023 Aug 30;621(7978):389–95. doi: 10.1038/s41586-023-06466-x (PMC10499599; doi:10.1038/s41586-023-06466-x)
Supplement: Supplementary file 2 — Reporting Summary [file 41586_2023_6466_MOESM2_ESM.pdf]

nature portfolio

Corresponding author(s): Hiroshi Ohno

Last updated by author(s): Jun 11, 2023

Reporting Summary

Nature Portfolio wishes to improve the reproducibility of the work that we publish. This form provides structure for consistency and transparency in reporting. For further information on Nature Portfolio policies, see our Editorial Policies and the Editorial Policy Checklist.

Statistics

For all statistical analyses, confirm that the following items are present in the figure legend, table legend, main text, or Methods section.

n/a	Confirmed	
☐	☒	The exact sample size (*n*) for each experimental group/condition, given as a discrete number and unit of measurement
☐	☒	A statement on whether measurements were taken from distinct samples or whether the same sample was measured repeatedly
☐	☒	The statistical test(s) used AND whether they are one- or two-sided<br>*Only common tests should be described solely by name; describe more complex techniques in the Methods section.*
☐	☒	A description of all covariates tested
☐	☒	A description of any assumptions or corrections, such as tests of normality and adjustment for multiple comparisons
☐	☒	A full description of the statistical parameters including central tendency (e.g. means) or other basic estimates (e.g. regression coefficient) AND variation (e.g. standard deviation) or associated estimates of uncertainty (e.g. confidence intervals)
☐	☒	For null hypothesis testing, the test statistic (e.g. *F*, *t*, *r*) with confidence intervals, effect sizes, degrees of freedom and *P* value noted<br>*Give P values as exact values whenever suitable.*
☒	☐	For Bayesian analysis, information on the choice of priors and Markov chain Monte Carlo settings
☒	☐	For hierarchical and complex designs, identification of the appropriate level for tests and full reporting of outcomes
☐	☒	Estimates of effect sizes (e.g. Cohen's *d*, Pearson's *r*), indicating how they were calculated

Our web collection on statistics for biologists contains articles on many of the points above.

Software and code

Policy information about availability of computer code

Data collection	Microsoft Excel 2016.
Data analysis	R (v4.1.1), R studio (v1.4.1717), R packages "psych" (v 2.1.6), "ppcor" (v 1.1), "Rfit" (v 0.24.2), "QuantPsyc" (v 1.5), "lme4" (v 1.1-27.1), "mRMRe" (v 2.1.2.1), "pROC" (v1.17.0.1), "caret" (v6.0-88), "ccrepe" (v1.28.0), "pheatmap" (v1.0.12), "mediation" (v 4.5.0), "WGCNA" (v1.72-1), "KEGGREST" (v 1.32.0), "vegan" (v2.6-4), and Cytoscape (v 3.7.0).

For manuscripts utilizing custom algorithms or software that are central to the research but not yet described in published literature, software must be made available to editors and reviewers. We strongly encourage code deposition in a community repository (e.g. GitHub). See the Nature Portfolio guidelines for submitting code & software for further information.

Data

Policy information about availability of data

All manuscripts must include a data availability statement. This statement should provide the following information, where applicable:

- Accession codes, unique identifiers, or web links for publicly available datasets
- A description of any restrictions on data availability
- For clinical datasets or third party data, please ensure that the statement adheres to our policy

Raw sequence data are deposited at the DNA Data Bank of Japan's BioProject (https://www.ddbj.nig.ac.jp/bioproject/index-e.html) under accession number PRJDB11444. Raw metabolomic data are deposited at the RIKEN DROP Met (http://prime.psc.riken.jp/menta.cgi/prime/drop_index) under index number DM0037. Raw CAGE sequencing data are deposited at the Japanese Genotype-phenotype Archive of National Bioscience Database Center (https://humandbs.biosciencedbc.jp/en/) under accession number JGAS000569. Following publicly available databases were used in this study: Ribosomal Database Project (http://rdp.cme.msu.edu/), CORE (http://microbiome.osu.edu/), a reference genome sequence database obtained from the NCBI FTP site (ftp://ftp.ncbi.nih.gov/

genbank/, December 2011), UCLUST (http://www.drive5.com/), the KEGG ORTHOLOGY database (https://www.genome.jp/kegg/ko.html), glycoside hydrolase family classification in the CAZy database (http://www.cazy.org/Glycoside-Hydrolases.html), the Inflammatory Bowel Disease Multi'omics Database (https://ibdmdb.org/), and the Human Gene Atlas database associated with Enrichr (https://maayanlab.cloud/Enrichr/).

Field-specific reporting

Please select the one below that is the best fit for your research. If you are not sure, read the appropriate sections before making your selection.

☒ Life sciences ☐ Behavioural & social sciences ☐ Ecological, evolutionary & environmental sciences

For a reference copy of the document with all sections, see nature.com/documents/nr-reporting-summary-flat.pdf

Life sciences study design

All studies must disclose on these points even when the disclosure is negative.

Sample size	No sample-size calculation was performed. The sample size in human study was determined based on previous metagenomic studies showing microbial signatures of diabetic patients (Qin, J. et al. Nature 2012, Karlsson, F. H. et al. Nature 2013). The sample size in experiments involving animals and bacterial culture was determined to be adequate based on the magnitude and consistency of measurable differences between groups based on previous reports and our preliminary experiments.
Data exclusions	One mouse with unsuccessful intravenous insulin injection was removed (Extended Data Fig. 9); otherwise no sample was removed from the experiments.
Replication	No replication in our human cohort, although the results were partly validated by other cohorts (TwinsUK and HMP2). All animal experiments were replicated a minimum of two to three times, yielding consistent results. The hyperinsulinemic euglycemic clamp test (Extended Data Fig. 9) was conducted once to validate the findings of insulin tolerance tests, which were repeated three times and yielded consistent results. The bacterial culture analyses were conducted twice and yielded similar results.
Randomization	The human participants were not randomized since this was a cross-sectional study. All of analyzed mice were randomly assigned to the groups, and they were age- and sex-matched (6 weeks of age, male).
Blinding	No blinding in the human sample analysis and animal experiments since these did not depend on investigator's observation and subjectivity.

Reporting for specific materials, systems and methods

We require information from authors about some types of materials, experimental systems and methods used in many studies. Here, indicate whether each material, system or method listed is relevant to your study. If you are not sure if a list item applies to your research, read the appropriate section before selecting a response.

Materials & experimental systems

n/a	Involved in the study	
☒	☐	Antibodies
☒	☐	Eukaryotic cell lines
☒	☐	Palaeontology and archaeology
☐	☒	Animals and other organisms
☐	☒	Human research participants
☒	☐	Clinical data
☒	☐	Dual use research of concern

Methods

n/a	Involved in the study	
☒	☐	ChIP-seq
☒	☐	Flow cytometry
☒	☐	MRI-based neuroimaging

Animals and other organisms

Policy information about studies involving animals; ARRIVE guidelines recommended for reporting animal research

Laboratory animals	C57BL/6 mice (6 weeks of age, male) were purchased from CLEA Japan and maintained under a conventional animal facility at Yokohama City University and RIKEN Yokohama Branch. The Yokohama City University animal facility is maintained in a 12-hour light and dark cycle at 24 ± 1.5°C and 55 ± 10% humidity. The RIKEN animal facility is maintained in a 12-hour light and dark cycle at 23 ± 2ºC and 50 ± 10% humidity.
Wild animals	No wild animals were used in this study.
Field-collected samples	No field-collected samples were used in this study.
Ethics oversight	All experimental procedures were approved by the Institutional Animal Care and Use Committee of the Yokohama City University and RIKEN and performed in accordance with the institutes' guidelines.

Note that full information on the approval of the study protocol must also be provided in the manuscript.

Human research participants

Policy information about studies involving human research participants

Population characteristics	The study participants were recruited from 2014 to 2016 during their annual health check-ups at University of Tokyo Hospital (Tokyo, Japan). The individuals included both male and female Japanese aged from 20 to 75 years old. The exclusion criteria are as follow: Established diagnosis of diabetes, routine use of medications for diabetes and/or intestinal diseases, use of antibiotics within two weeks prior to sample collection, and those who lost three kg of body weight in three months prior to sample collection.
Recruitment	The study participants were widely recruited via brochures and posters before and at health-checkups. To normalize the participants' clinical characteristics, we planned to recruit roughly 100 normal, 100 obese (BMI ≥ 25, based on the Japanese definition), and 100 prediabetic (FBG ≥ 110 mg/dL and/or HbA1c ≥ 6.0 %) individuals based on their clinical data, and stopped recruiting when the number of participants almost reached the goal. The sample size was determined based on previous metagenomic studies showing microbial signatures of diabetic patients. We enrolled 112, 100, and 101 individuals for normal, obese, and prediabetic groups, respectively. Among them, two individuals withdrew from the study after enrollment, and five individuals did not provide fecal specimens. Given that we recruited participants from health-checkups, who are typically regarded as individuals with a high level of health consciousness, there is a possibility of potential selection bias.
Ethics oversight	The study was approved by the institutional review board of RIKEN and The University of Tokyo and performed in accordance with the institutes' guidelines.

Note that full information on the approval of the study protocol must also be provided in the manuscript.
